# Panels of mRNAs and miRNAs for decoding molecular mechanisms of Renal Cell Carcinoma (RCC) subtypes utilizing Artificial Intelligence approaches

**DOI:** 10.1038/s41598-022-20783-7

**Published:** 2022-09-30

**Authors:** Seyed Mahdi Hosseiniyan Khatibi, Mohammadreza Ardalan, Mohammad Teshnehlab, Sepideh Zununi Vahed, Saeed Pirmoradi

**Affiliations:** 1 Clinical Research Development Unit of Tabriz Valiasr Hospital, Tabriz University of Medical Sciences, Tabriz, Iran; 2 Kidney Research Center, Tabriz University of Medical Sciences, Daneshgah Street, Tabriz, Postal Code 51665118 Iran; 3 Rahat Breath and Sleep Research Center, Tabriz University of Medical Science, Tabriz, Iran; 4 Department of Electric and Computer Engineering, K.N. Toosi University of Technology, Tehran, Iran

**Keywords:** Machine learning, Cancer genomics

## Abstract

Renal Cell Carcinoma (RCC) encompasses three histological subtypes, including clear cell RCC (KIRC), papillary RCC (KIRP), and chromophobe RCC (KICH) each of which has different clinical courses, genetic/epigenetic drivers, and therapeutic responses. This study aimed to identify the significant mRNAs and microRNA panels involved in the pathogenesis of RCC subtypes. The mRNA and microRNA transcripts profile were obtained from The Cancer Genome Atlas (TCGA), which were included 611 ccRCC patients, 321 pRCC patients, and 89 chRCC patients for mRNA data and 616 patients in the ccRCC subtype, 326 patients in the pRCC subtype, and 91 patients in the chRCC for miRNA data, respectively. To identify mRNAs and miRNAs, feature selection based on filter and graph algorithms was applied. Then, a deep model was used to classify the subtypes of the RCC. Finally, an association rule mining algorithm was used to disclose features with significant roles to trigger molecular mechanisms to cause RCC subtypes. Panels of 77 mRNAs and 73 miRNAs could discriminate the KIRC, KIRP, and KICH subtypes from each other with 92% (F1-score ≥ 0.9, AUC ≥ 0.89) and 95% accuracy (F1-score ≥ 0.93, AUC ≥ 0.95), respectively. The Association Rule Mining analysis could identify miR-28 (repeat count = 2642) and CSN7A (repeat count = 5794) along with the miR-125a (repeat count = 2591) and NMD3 (repeat count = 2306) with the highest repeat counts, in the KIRC and KIRP rules, respectively. This study found new panels of mRNAs and miRNAs to distinguish among RCC subtypes, which were able to provide new insights into the underlying responsible mechanisms for the initiation and progression of KIRC and KIRP. The proposed mRNA and miRNA panels have a high potential to be as biomarkers of RCC subtypes and should be examined in future clinical studies.

## Introduction

Renal Cell Carcinoma (RCC) possesses the 15th rank among frequent cancers, according to the GLOBOCAN report^1^. RCC comprises 3% of all malignant neoplastic cases in adults and approximately 90% of malignant kidney tumors^2^. Based on the American Cancer Society estimation, 1 in 46 men and 1 in 80 women will be diagnosed with RCC during their life. Also, the 5-year survival rate of patients with RCC is 73.7%^3^. Smoking, obesity, and hypertension are important risk factors that can affect RCC occurance^4^. The incident and mortality rate of RCC highlight the importance of screening programs to develop reliable biomarkers for early detection of its subtypes^5^.

The histological subtypes of renal cancer include; clear-cell RCC (ccRCC or KIRC, 60–80% of all patients), papillary RCC (pRCC or KIRP, 10–15%), chromophobe RCC (chRCC or KICH, 5–10%), and other rare subtypes (< 1%)^6^. KIRC is identified by mutations in the *VHL* (von Hippel–Lindau) gene and the loss of chromosome 3p. The clinical severity of KIRC is higher compared with KIRP and KICH subtypes (5-year survival rate of 55–60%, 80–90%, and 90%, respectively)^7^ due to the lack of effective biomarkers for earlier detection. In the early stages, KIRC is frequently asymptomatic and 25–30% of patients are often diagnosed at metastasis status; hence, presenting a high mortality rate^8^. KIRP is characterized by the loss of chromosome 9p and trisomy of chromosomes. In KIRP, a few subgroups of patients have a satisfactory treatment outcome, while a wide range of patients needs to develop promising treatment strategies^9,10^. Small incidentally detected kidney masses have a major diagnostic dilemma since a section of them can be benign and managed conservatively^11^. A lower-risk RCC, KICH, is identified by the loss of chromosomes and can pose a slight risk to a patient if cautiously treated with routine surveillance rather than surgery^11^. KICH has enormous potential to be diagnosed earlier compared to other RCC subtypes due to its variable long-term outcome^12^. Due to these distinct clinical and biological behaviors, differentiating and accurate detection of RCC subtypes via non-invasive and precise biomarkers confidently help physicians perform an appropriate therapeutic decision. This could be different from total and partial nephrectomy or even close follow-ups.

It has been shown that candidate biomarkers are not reliable for the diagnosis and prognosis of different RCC subtypes^13–16^ and none of them can be employed in clinical application^17^. Furthermore, computed tomography (CT) and abdominal ultrasound have problems including high costs and low sensitivity in the diagnosis of small tumors^4,18^. Thus, the development of non-invasive and accurate screening for different types of RCC is a necessity. Comprehensive understanding of the molecular mechanisms of RCC subtypes is the main challenge in RCC research to identify novel and reliable molecular biomarkers.

This study was designed to introduce panels of mRNAs and miRNAs for discriminating different subtypes of RCC and detect remarkable molecules that have significant pathological roles in RCC subtypes through machine learning and artificial intelligence approaches. Firstly, we implemented reading and pre-processing of the RNA-sequencing data. Next, to identify candidate features (mRNAs and miRNAs), we applied feature selection based on filter and graph algorithms. Then, to evaluate selected candidate features, we employed a deep learning model to classify the subtypes of RCC. Finally, an association rule mining algorithm was used for detecting remarkable features which play significant roles in firing molecular mechanisms to cause RCC subtypes.

## Material and method

### Material

In this study, the renal cell carcinoma data, including mRNA, miRNA, and clinical data were downloaded from the GDC portal (https://portal.gdc.cancer.org) provided by The Cancer Genome Atlas (TCGA) dataset^19^. Downloaded data were utilized to study the molecular mechanism of RCC subtypes, including ccRCC (KIRC), pRCC (KIRP), and chRCC (KICH). In the TCGA renal cell carcinoma project, 60,482 mRNAs expression and 1,881 miRNAs expression were reported for each patient. mRNA expression and clinical data were provided for 611 ccRCC patients, 321 pRCC patients, and 89 chRCC patients. Moreover, miRNA expression and clinical data were reported for 616 patients in the ccRCC subtype, 326 patients in the pRCC subtype, and 91 patients in the chRCC subtype. Information on each subtype is presented in Table 1 in more detail. This study was conducted according to the principles of the Declaration of Helsinki (2013).

**Table 1:** Subtypes information of RCC in detail.

Genomic Data	Number of patients with Renal Cell Carcinoma (RCC)		
	pRCC (KIRP)	ccRCC (KIRC)	chRCC (KICH)
mRNA	321	611	89
miRNA	326	616	91

### Method

**Figure 1:**
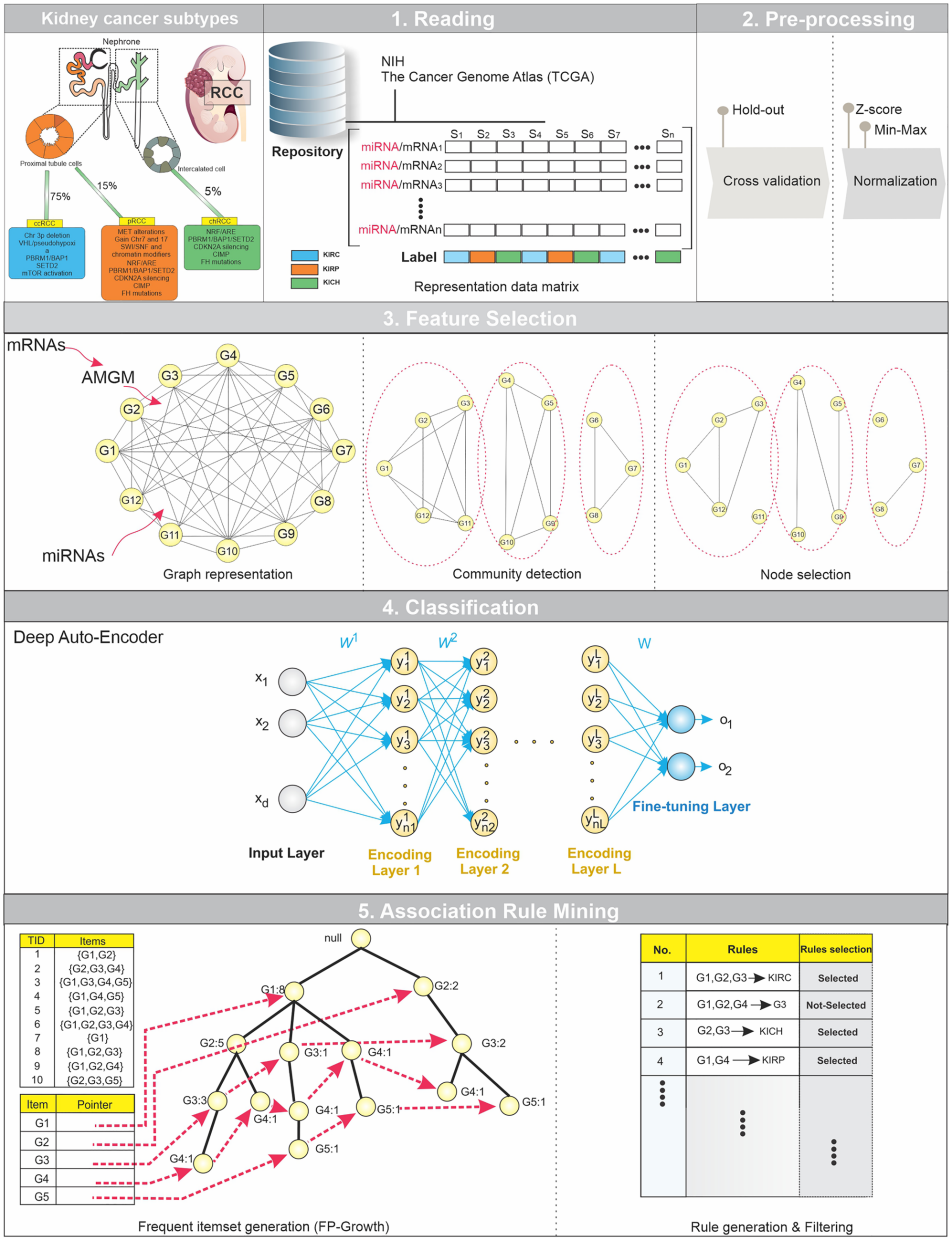
The overview of the proposed method: Five main steps, including reading, preprocessing, feature selection, classification, and association rule mining were applied to miRNA and mRNA expression data. (1) In the reading step, each dataset was downloaded from the TCGA repository. (2) The preprocessing step includes two sub-steps, cross-validation, and normalization. (3) The feature selection step contains two sub-steps, the filter method based on AMGM value for mRNA data and the graph-based method, in which candidate miRNAs/mRNAs with more relevance to RCC subtypes were selected. (4) A deep classifier model was utilized to evaluate the discrimination power of selected miRNAs/mRNAs. (5) The Association Rule Mining method discovers the hidden relationship between selected miRNAs/mRNAs and RCC subtypes in the first level and the complex relationship among selected miRNAs/mRNAs in the second level.

The proposed method contained four main steps: reading and preprocessing, feature selection, classification, and filtering, as shown in Fig. 1. In the 1st and 2nd steps, miRNA, mRNA, and clinical data were preprocessed using the necessary preprocessing methods. In the 3rd step, irrelevant and redundant features (mRNAs and miRNAs) were removed from the data using the proposed algorithms. In the 4th step, the deep classifier model was applied to distinguish defined groups based on the candidate features obtained in the previous step. In the 5th step, the association rule mining algorithm was employed for identifying the predominant features among candidate features playing a crucial role in each group.

### Reading and preprocessing

miRNA and mRNA data were interpreted as a matrix composed of rows and columns, with approximately equal numbers of rows/columns; 1033/1881 and 1021/60,482, respectively. Next, redundant features were removed from the data, including 2256 mRNAs from 60,482 mRNAs and 336 miRNAs from 1881 miRNAs. Then, the hold-out cross-validation method was utilized to split data into three parts; training, validation, and test data by considering 70%, 10%, and 20% contributions, respectively. Finally, the min–max was used to scale mRNAs and miRNAs values in the [0 1] range. We applied z-score [Eq. (1)] for normalization and min–max [Eq. (2)] in feature selection and classification steps based on the need of the proposed algorithms in these steps, respectively.

1 $$y= \frac{x-\mu }{\sigma }$$

2 $$y= \frac{1}{Max-Min}(x-Min)$$

In Eqs. (1) and (2), $y$ and $x$ are the normalized and raw feature values, respectively. The µ, σ, Min, and Max are defined as mean value, standard deviation, minimum value, and maximum value of x, sequentially.

### Feature selection

In feature selection, we applied a new graph-based method for feature selection in mRNA and miRNA data separately^20^. Therefore, a suitable subset of candidate features was identified using the proposed algorithms. In miRNA data, a graph-based algorithm extracted the suitable subset of candidate features among 1545 features. However, in mRNA data, we first utilized the filter-based method for primary feature selection due to the existence of 58,226 features that could cause a high computational cost in the graph-based algorithm. Thus, the filter-based approach reduced the mRNA data dimension from 58,226 to 1000, and a graph-based algorithm extracted the suitable subset of candidate features among 1000 features. In the following, the proposed algorithms are specified in more detail.

### Filter method

Filter-based methods interpret the feature selection process by calculating importance measures for each feature separately. These algorithms do not apply any evaluation tool such as a classifier and are called classifier-independent techniques. Filter-based methods have low computational and time costs; however, they do not consider features interactions in the feature selection process. In this sub-step, we applied the filter-based method for primary feature selection in mRNA data. Filter-based method help to remove some irrelevant features and reduce the dimensionality for use in the graph-based process. Many filter methods have been introduced and utilized in various data with different domains, but a few are suitable for high dimension and low sample size (HDLSS) data^21^. In this regard, we used the $AMGM$ measure to evaluate the importance of features. The $AMGM$ filter has illustrated its powerful potential in HDLSS data^22^. The $AMGM$ measure is calculated by Eq. (3).

3 $${AMGM{:} R}_{i}= \frac{{AM}_{i}}{{GM}_{i}} \in [1, +\infty )$$

In Eq. (3), the ${AM}_{i}$ and ${GM}_{i}$ are arithmetic mean, and the geometric mean of the ith feature are shown in Eqs. (4) and (5), respectively. ${R}_{i}$ presents a dispersion of the ith feature among all samples. The higher ${R}_{i}$ mentions a high dispersion and more relevant feature concerning defined phenotype. When ${R}_{i}$ is close to one, which means the i^th^ feature has low relevancy with a defined phenotype.

4 $${AM}_{i}= {\overline{x} }_{i}= \frac{1}{n} \sum_{i=1}^{n}{x}_{ij}$$

5 $${GM}_{i}= {(\prod_{i=1}^{n}{x}_{ij})}^\frac{1}{n}$$

If the i^th^ feature contains zero among reported values, then ${GM}_{i}=0$ based on Eq. (5) and $AMGM$ measure will be inefficient. The modified version of $AMGM$ is defined to avoid this problem based on Eq. (6). In the revised version, the exponential function was applied to features in the numerator and denominator of the $AMGM$ formula.

6 $$AMGM{:} {R}_{i}= \frac{\frac{1}{n}\sum_{i=1}^{n}{\mathrm{exp}(x}_{ij})}{{(\prod_{i=1}^{n}{\mathrm{exp}(x}_{ij}))}^\frac{1}{n}}= \frac{1}{n \times \mathrm{exp}({\overline{x} }_{i})}\sum_{i=1}^{n}{\mathrm{exp}(x}_{ij})$$

### Graph method

Recently, graph-based methods are used for feature selection^20,23^. These methods display the search space as a graph using a graph representation of features. Then the principles of graph theory are employed for selecting the most relevant attributes. The graph-based method contains three sub-steps, including graph representation of feature space, community detection/graph clustering, and selecting the significant nodes in each cluster.

#### Graph representation

In the first phase, the feature set is mapped to the graph space. The graph is defined by $\mathrm{G }= <\mathrm{F},\mathrm{ E}>$, in which $F=\{{F}_{1},{F}_{2},\dots , {F}_{n}\}$ and $E=\{\left({F}_{i}, {F}_{j}\right): {F}_{i}, {F}_{j} \epsilon F\}$ is a set of nodes and edges, respectively. Each feature represents a node, and relevance between two features deputes an edge in the graph structure. In this work, the ${W}_{ij}$ is used to measure the relationship between two features ${F}_{i}$ and ${F}_{j}$, as shown in Eq. (7).

7 $${W}_{ij}= \left\{\begin{array}{ll}\beta \times Relevancy-\left(1-\beta \right)\times Redundancy & if i\ne j \\ 1 & otherwise\end{array}\right.$$

8 $$Relevancy= \frac{\mathrm{AMGM}({F}_{i}) +\mathrm{ AMGM}({F}_{j})}{2}$$

9 $$Redanduncy= \left|\frac{\langle {F}_{i},{F}_{j}\rangle }{\Vert {F}_{i}\Vert \times \Vert {F}_{j}\Vert }\right|=\mathrm{cos}({\theta }_{{F}_{i},{F}_{j}})$$

${W}_{ij}$ contains two parts, including relevancy and redundancy. In relevancy measure [Eq. (8)], $AMGM\left({F}_{i}\right)$ and $AMGM({F}_{j})$ are the $AMGM$ value of features ${F}_{i}$ and ${F}_{j}$, respectively. Unlike the Bakhshandeh et al. that used symmetric uncertainty measure^20^, we applied the $AMGM$ to measure relevancy; due to its high potential in HDLSS data. In redundancy measure, the similarity of two features is calculated based on the cosine similarity by Eq. (9), in which $\langle ,\rangle$ and $\Vert .\Vert$ are the inner product and Euclidean norm, respectively. Also, β ($0<\beta <1$) is the user-defined parameter that controls the potency of each part.

${\widehat{W}}_{ij}$ is the normalized relationship between two features in the range [0 1], calculated by the SoftMax scaling function, as shown in Eq. (10). We employed ${\widehat{W}}_{ij}$ as the weight of edge in the graph structure. In the SoftMax scaling, $\overline{W }$, $\sigma$ are the mean and standard deviation of weights, respectively.

10 $${\widehat{W}}_{ij}= \frac{1}{1+\mathrm{exp}(- \frac{{W}_{ij}-\overline{W}}{\sigma })}$$

#### Community detection

In the second phase, we applied a community detection algorithm to cluster graph space. The community detection algorithm performs clustering by finding groups of nodes that have high-density connectors internally. Nodes in the same community have high similarity properties. Community detection assists in better understanding sophisticated networks, such as genomic data networks. In recent years, researchers have introduced various community detection algorithms. Louvain algorithm is one of the fastest among them. Louvain algorithm performs the clustering process by maximizing the modularity objective function, in which the quality of partitions is compared, by the community detection process^24^. In addition, the Louvain algorithm is simple in the implementation phase.

In a graph with n nodes, the Louvain algorithm starts firstly with n communities by allocating each node to one community. The algorithm works based on a random selection of a node and transferring from its community to another one, then the gain modularity is calculated. This process is repeated until no improvement happens, and the algorithm will finish.

#### Node selection

In the third phase, we need to select the significant nodes in each community/cluster. In this regard, the Maximum Independent set (MIS) concept based on graph theory is utilized for node selection in subgraphs. A subset of the vertex set of a graph is independent if and only if it includes no pair of adjacent vertices. Identifying the independent set with the maximum size is an NP-hard optimization problem. It is doubtful that there exists an efficient algorithm for finding an MIS of a graph. In this study, we applied the proposed algorithm of R. Boppana et al. based on^25^ to get the MIS in each community/cluster. Also, we defined the adjacency matrix to employ in the R. Boppana algorithm. It is a Boolean graph matrix that is calculated by Eqs. (11) and (12). Where γ is the user-defined parameter ($0<\gamma <1$).

11 $$Adjacency \, Matrix={\left[{a}_{ij}\right]}_{n\times n}$$

12 $${a}_{ij}= \left\{\begin{array}{ll}1 & {\widehat{W}}_{ij}> \gamma and i\ne j \\ 0 & otherwise\end{array}\right.$$

### Classification

In this step, we applied a classifier to evaluate the candidate features selected in the previous step. High accuracy (or any user-defined measure) of classification can mark the success of the feature selection method in choosing the relevant attributes. Otherwise feature selection method cannot identify relevant features.

Employing this method, we constructed a self-organizing deep auto-encoder model to classify data based on candidate features. A self-organizing deep auto-encoder is a specific type of deep auto-encoder that can determine its structure automatically, including the number of neurons and layers^26^. The description details of the self-organizing deep auto-encoder are available in Supplementary Methods. First, the training process of the deep model and the model selection were performed by training and validation data, respectively. Next, the performance of the classification was estimated by employing test data. The accuracy, F_1_-score, and AUC-ROC were applied to evaluate the classifier performance, as shown in Eqs. (13)–(16).

13 $$Accuracy= \frac{TP+TN}{TP+FN+FP+TN}\times 100$$

14 $${F}_{1}-score= 2\frac{Precision\times Recall}{Precision+Recall}$$

15 $$Recall=\frac{TP}{TP+FN}$$

16 $$Precision=\frac{TP}{TP+FP}$$

where TP, TN, FP, and FN are True Positive, True Negative, False Positive, and False Negative, respectively. AUC-ROC is the area under the Receiver Operating Characteristic (ROC) curve.

### Association rule mining

Association Rule Mining can extract efficient associations among data items. An association rule is defined in $A\to C$ form, in which A and C are Antecedent and Consequent, respectively. If we consider A as the feature(s) and C as the feature(s)/user-defined phenotype, association rule mining can show interesting dependency between feature(s)-feature(s) and feature(s)-phenotype. Therefore, it is reasonable to apply the association rule mining-based method to identify important features among candidate features. In this regard, we applied the association rule mining algorithm to candidate features, which was obtained in the feature selection step. In the following, some principal concepts of association rule mining are introduced. Support, Confidence, and Lift are three important measures in association rule mining. Let $I=\{{i}_{1}, {i}_{2}, \dots ,{i}_{d}, y\}$ be a set of items, $D=\{{d}_{1}, {d}_{2}, \dots , {d}_{n}\}$ be a dataset of n instances, $F=\{{f}_{1}, {f}_{2}, \dots , {f}_{m}\}$ be the features space with m features, and $Y=\{0, 1\}$ be the user-defined phenotype. The ${d}_{i}$ can be presented as a tuple $({X}_{i}, {y}_{i})$, where ${X}_{i}\in {f}_{1}\times {f}_{2}\times \dots \times {f}_{m}$ and ${y}_{i}\in Y$. Also, $A\to C$ is an association rule, where $A\subset I$, $C\subset I$, and $A\cap B= \varphi$. The support of rule $A\to C$ is the probability of instances containing both A and C, as shown in Eq. (17). Support evaluates the rule's usefulness.

17 $$Support\left(A\to C\right)= \frac{support(A\cup C)}{n}$$

In Eq. (17), $Support\left(A\right)=|\left\{{d}_{i}\right|A\subseteq {X}_{i}, {d}_{i}\in D\}|$ is the number of instances that includes the $i$ itemset. The confidence of rule $A\to C$ is the probability, which shows the frequency of cases with $C$ among all samples containing $A$, as shown in Eq. (18). Confidence estimates the rule's certainty.

18 $$Confidence\left(A\to C\right)=P\left(C|A\right)= \frac{support(A\cup C)}{support(A)}$$

The Lift of rule $A\to C$ determines the dependency between the occurrence of itemset $A$ and $C$. When the Lift value is more (less) than one, the occurrence of $A$ is positively (negatively) correlated with the occurrence of $C$. If the Lift value is equal to one, then $A$ and $C$ are independent. The Lift value is shown in Eq. (19).

19 $$Lift\left(A\to C\right)= \frac{P(A\cup C)}{P\left(A\right)P(C)}$$

#### FP-growth algorithm

Association rule mining mainly contains two phases: Frequent itemset generation and Rule generation. In the Frequent itemset generation, the algorithm generates all itemsets iteratively, then itemset that its support count is more than the min_support (user-defined threshold) reported as a frequent itemset. In the rule generation, association rules are made based on frequent itemsets. Agrawal et al. introduced the Apriori algorithm as one of the first association analysis algorithms, in which the property of frequent itemset is applied successfully by R. Agrawal^27^. In recent years many researchers have proposed many improved algorithms, such as FP-Growth^28,29^, Apriori-Hybrid^30^, Fuzzy association rule^31^, etc.

In this study, we utilized the FP-Growth algorithm for association analysis. In terms of computational, storage space, and time, the FP-Growth is one of the best algorithms for association analysis due to one-time searching itemset space. The pseudo-code of the FP-Growth algorithm is shown in Tables 1 and 2 in Supplementory method in more detail.

## Results

We executed the following steps to the miRNA and mRNA data (Fig. 1). In the feature selection step, we applied a graph-based method to the miRNA data. First, the graph was constructed with 1545 nodes and 1,194,285 edges, and the weights of the edges were calculated based on the $AMGM$ and cosine similarity measures. Next, the Louvain algorithm identified 40 communities/clusters from each; and finally, 73 candidate miRNAs were selected among communities/clusters using the MIS algorithm. Moreover, β and γ, the parameters of the graph-based method were set to 0.6 and 0.3, respectively.

**Figure 2:**
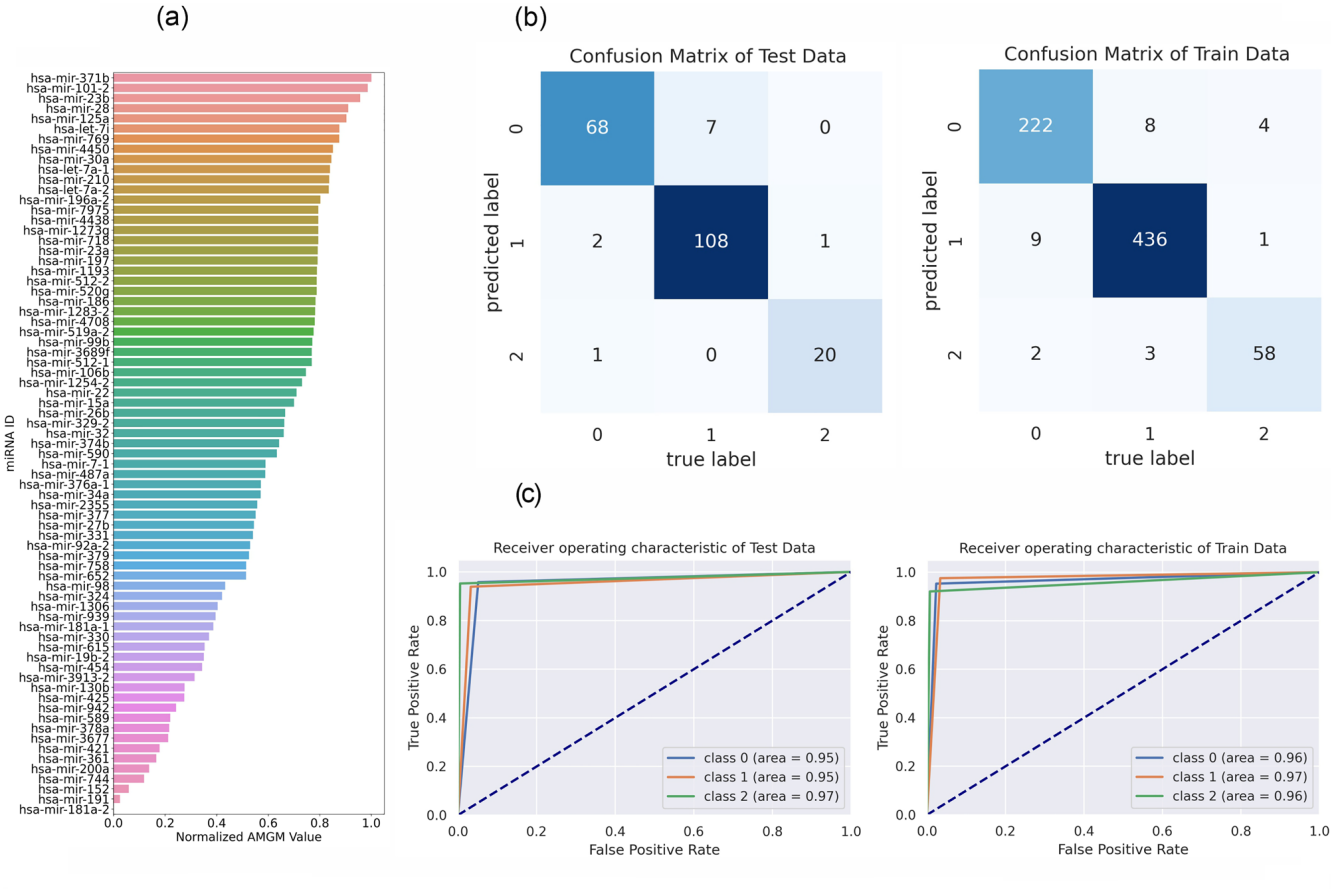
The performance of feature selection and classification steps in the miRNA data. (**a**) Bar plot of candidate miRNAs (73) based on normalized AMGM values. (**b**) Confusion matrix of training and test data, in which 0, 1, and 2 are pointed to KIRP, KIRC, and KICH groups, respectively. (**c**) Receiver Operating Characteristic (ROC) curve of training and test data. The Area under the Curve of Receiver Operating Characteristic (AUC-ROC) is reported for each curve.

**Figure 3:**
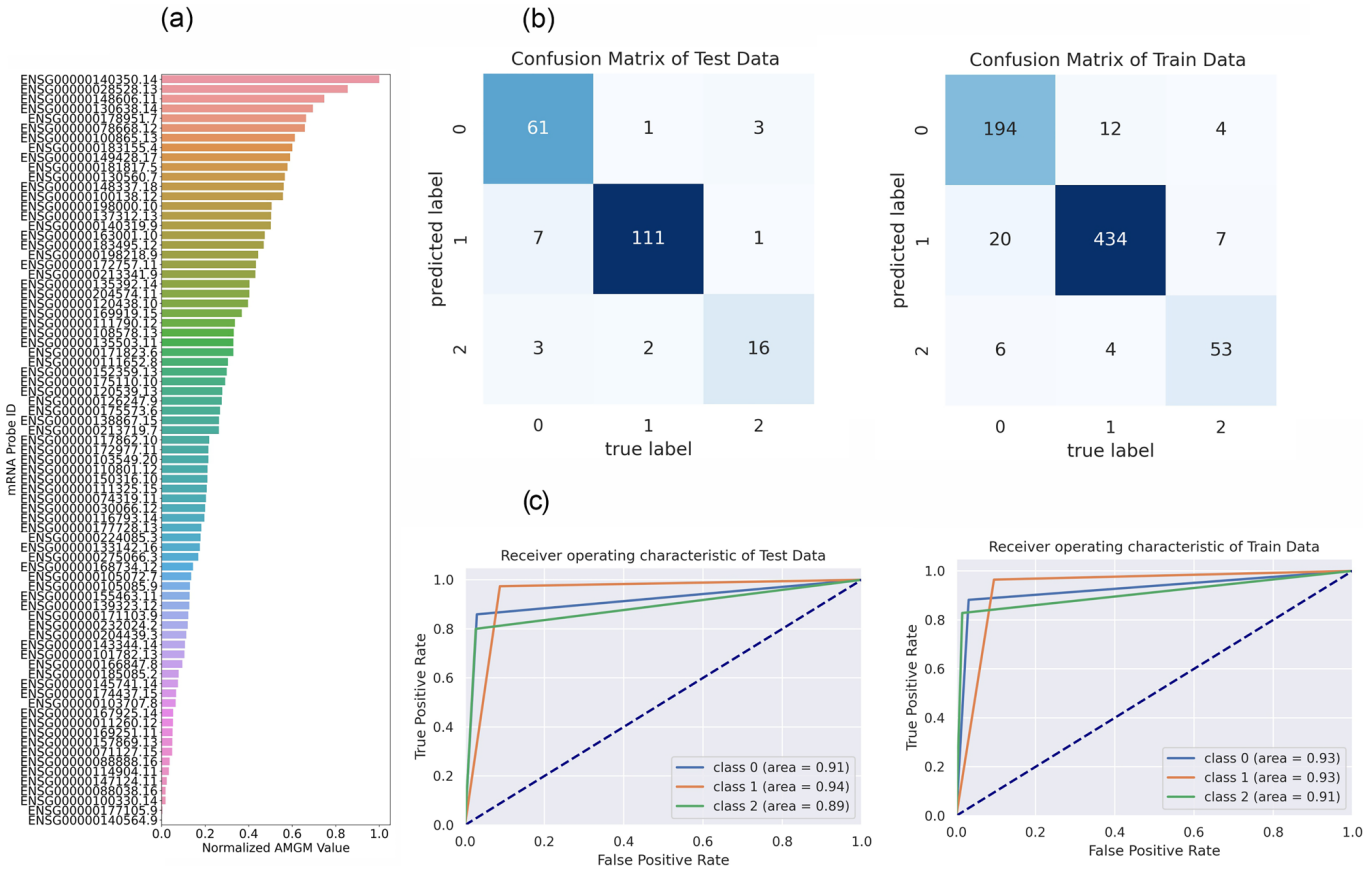
The presentation of feature selection and classification steps in the mRNA data. (**a**) Candidate mRNAs (77) are presented by Bar plot based on normalized AMGM values. (**b**) Training and test data confusion matrix. (**c**) ROC curve of training and test data. The AUC-ROC is reported for each curve. 0, 1, and 2 are pointed to KIRP, KIRC, and KICH groups, respectively. *AUC-ROC* Area under the Curve of Receiver Operating Characteristic.

In mRNA data, first, we used the filter method based on the $AMGM$ measure to remove some irrelevant features. In the primary feature selection, we selected 1000 top features with the highest $AMGM$ value, then we employed a graph-based method for the mRNA data. The graph was composed of 1000 nodes and 504,510 edges. Next, community detection of the mRNA network graph was performed by the Louvain algorithm that identified 73 communities/clusters. Finally, 77 candidate mRNAs were selected from communities/clusters using the MIS algorithm where β and γ parameters of the graph-based method, were set to 0.5 and 0.3, respectively. The list of 73 candidate miRNAs and 77 candidate mRNAs are reported in Supplementary Tables 1 and 2, respectively. Also, these candidate mRNAs and miRNAs are illustrated in Figs. 2a and 3a based on their sorted $AMGM$ measure.

We applied the classifier to evaluate the discrimination power of candidate features (mRNAs/miRNAs) among RCC subtypes. In this regard, the self-organizing deep auto-encoder was utilized for performing classification tasks. The accuracy, F_1_-score, and AUC of mRNA and miRNA data are reported individually in Table 2. Also, the confusion matrix and ROC curve are illustrated for miRNA and mRNA data in Figs. 2 and 3, respectively. Results indicated that machine learning-derived mRNAs (Accuracy = 92%) and miRNAs (Accuracy = 95%) panels could significantly distinguish these subtypes from each other with high accuracy.

**Table 2:** The performance metrics for the classification step.

	miRNA			mRNA		
	Train	Test	Validation	Train	Test	Validation
Accuracy (%)	96	**95**	96	93	**92**	91
F_1_-score (KIRP)	0.95	0.93	0.93	0.90	0.90	0.89
F_1_-score (KIRC)	0.98	0.96	0.98	0.95	0.95	0.95
F_1_-score (KICH)	0.92	0.95	0.92	0.83	0.78	0.75
AUC (KIRP)	0.96	0.95	0.94	0.93	0.91	0.91
AUC (KIRC)	0.97	0.95	0.96	0.93	0.94	0.94
AUC (KICH)	0.95	0.97	0.92	0.91	0.89	0.80

Significant values are in bold.

*AUC* Area under the Curve, *KIRC* kidney renal clear cell carcinoma, *KIRP* kidney renal papillary cell carcinoma, *KICH* kidney chromophobe carcinoma.

In similar studies, all subtypes, including ccRCC, pRCC, chRCC, WT (Wilms tumor), and RT (Rhabdoid tumor), were classified based on selected miRNAs in the feature selection process. However, these subtypes are different in cancer nature and patient type. The ccRCC, pRCC, and chRCC are common adult kidney cancer. In contrast, WT and RT are common pediatric kidney cancer. Thus, feature selection and classification of all subtypes may lead to missing information related to pediatric kidney cancer subtypes. In addition, the comparison classification accuracy of similar studies with the proposed method will be difficult due to this difference. Nevertheless, the accuracy of the applied methods is illustrated in Table 3. Also, we do not find any similar studies related to ccRCC, pRCC, and chRCC subtypes based on mRNA data for comparison.

**Table 3:** Comparison of classification accuracy based on miRNA data.

Methods	Accuracy (%)	Ref.
One way ANOVA + logistic regression (all subtypes)	86	^32^
One way ANOVA + decision tree (all subtypes)	87	^33^
Resampling + NCA + LSTM (all subtypes)	95.5	^34^
AMGM + deep neuro-fuzzy (all subtypes)	93.2	^35^
Proposed method (ccRCC, pRCC, and chRCC subtypes)	**95**	–

Significant values are in bold.

In the association rule mining process, candidate features were scaled into the [0 1] range using min–max normalization. Next, candidate features (mRNAs/miRNAs) were discretized into three categories, including low, medium, and high levels, so that the number of miRNA and mRNA items were equal to 212 and 233, respectively. Then, the FP-Growth algorithm was applied to generate frequent itemsets and association rules. Parameters of the algorithm, including min-support (frequent itemset), max-length (maximum length of frequent itemset), and lift (association rule), were set to 0.1, 4, and 1.1, sequentially.

**Figure 4:**
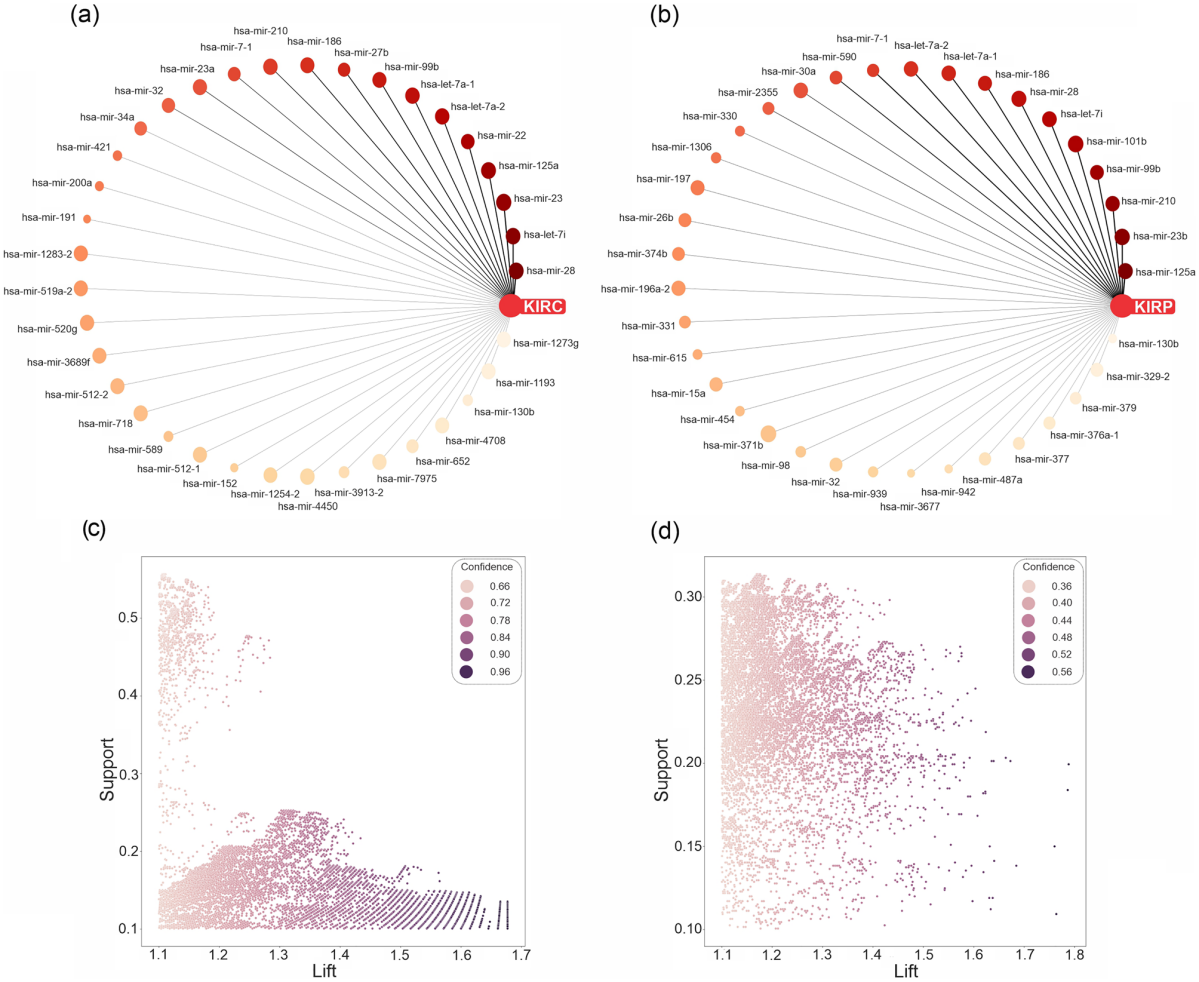
The relationship and specification of the KIRC/KIRP association rules in the miRNA data. Graph network of top miRNAs with high repeat count in (**a**) KIRC and (**b**) KIRP association rules. The most frequent miRNAs were displayed with the weight of the edge based on repeat counts. Also, the AMGM value of miRNAs was illustrated with the size of the node. Strength distribution of (**c**) KIRC and (**d**) KIRP association rules according to their support, lift, and confidence. *KIRC* kidney renal clear cell carcinoma, *KIRP* kidney renal papillary cell carcinoma, *KICH* kidney chromophobe carcinoma.

**Figure 5:**
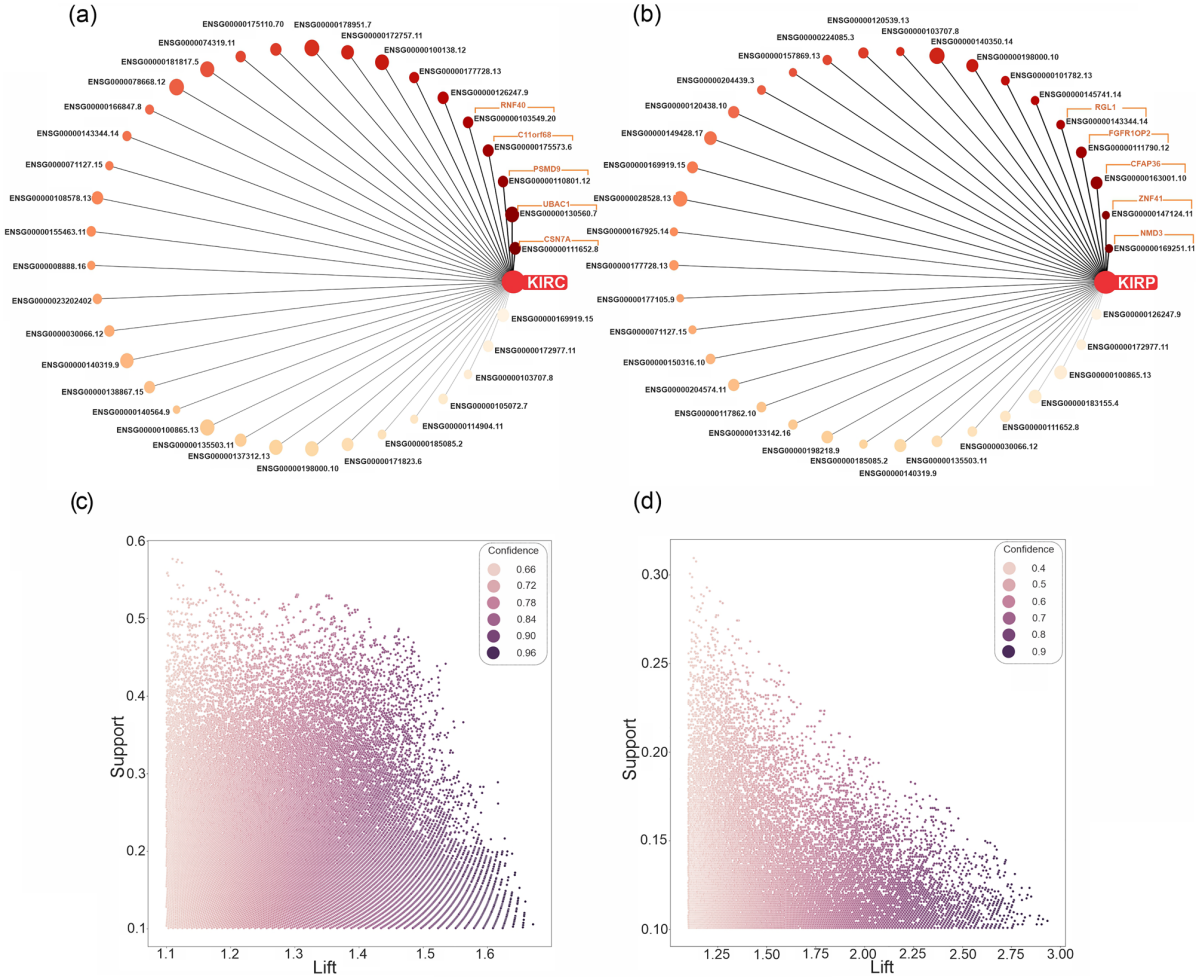
The KIRC/KIRP association rules in the mRNA data. Graph network of (**a**) KIRC and (**b**) KIRP association rules of top mRNAs with a high repeat count. The most frequent mRNAs were displayed with the weight of the edge based on repeat counts. The mRNAs’ AMGM value is illustrated with the size of the node. Strength distribution of (**c**) KIRC and (**d**) KIRP association rules according to their support, lift, and confidence. *KIRC* kidney renal clear cell carcinoma, *KIRP* kidney renal papillary cell carcinoma, *KICH* kidney chromophobe carcinoma.

To discover patterns, association rules that consequently were equal to KIRC/KIRP/KICH were selected. In the miRNA data, the number of association rules related to KIRC/KIRP was equal to 27,635/23,198. Moreover, in the mRNA data, the number of association rules related to KIRC/KIRP was equal to 94,354/28,956. Due to the lack of samples in the KICH subtype, frequent itemsets and related association rules were not generated; the support count of itemsets was less than the min-support threshold. In the antecedent part of the selected association rules, mRNAs and miRNAs with the highest repeat count were considered significant features in each RCC subtype. In Figs. 4a,b and 5a,b, significant miRNAs and mRNAs are shown as a graph network based on repeat count in KIRC/KIRP rules. Moreover, the strength distribution of KIRC/KIRP association rules according to their support, lift, and confidence is illustrated in Figs. 4c,d and 5c,d for miRNA and mRNA, respectively. In this regard, mRNAs and miRNAs were identified based on sorted repeat count (Supplementary Table 3). We hypothesized that these top features with the highest repeat counts may play a fundamental role in the pathogenesis of specific subtypes.

**Figure 6:**
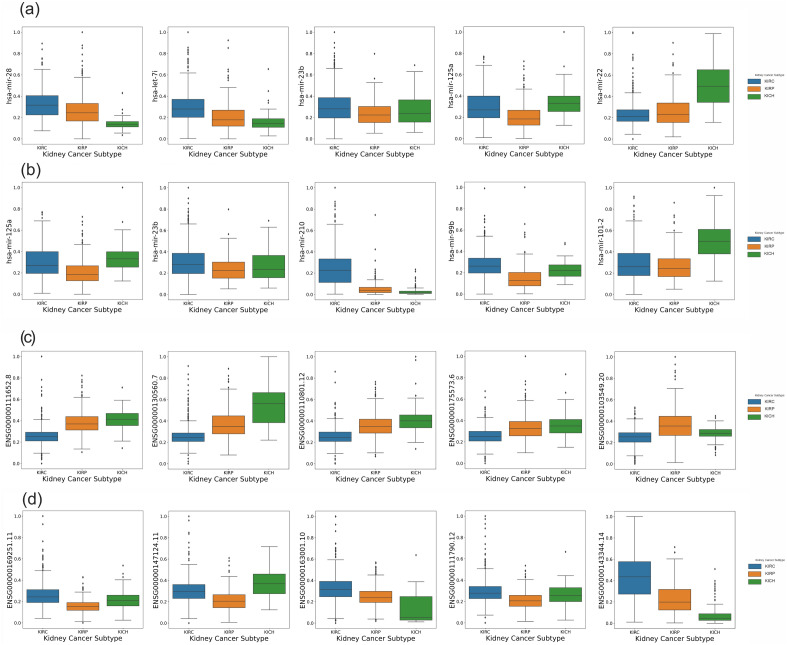
The selected miRNA and mRNA are based on the association rule mining step. Box plot of selected miRNAs for (**a**) KIRC and (**b**) KIRP groups. Box plot of selected mRNAs for (**c**) KIRC and (**d**) KIRP groups. Expression values of miRNAs and mRNAs were normalized in the range of 0 to 1 by the min–max method. *KIRC* kidney renal clear cell carcinoma, *KIRP* kidney renal papillary cell carcinoma, *KICH* kidney chromophobe carcinoma.

Box plots of five top miRNAs and mRNAs in KIRC and KIRP subtypes are illustrated in Fig. 6. The comparison of medians, interquartile ranges, and whiskers in box plots of significant miRNAs and mRNAs demonstrated a notable difference in all RCC subtypes. The pair plot of five top miRNAs and mRNAs of KIRC/KIRP rules are illustrated in Supplementary Figs. 1c,d and 2c,d, respectively. Moreover, the correlation of the top ten miRNAs and mRNAs of KRIC/KIRP rules is shown in Supplementary Figs. 1a,b and 2a,b, respectively based on the Spearman correlation.

**Figure 7:**
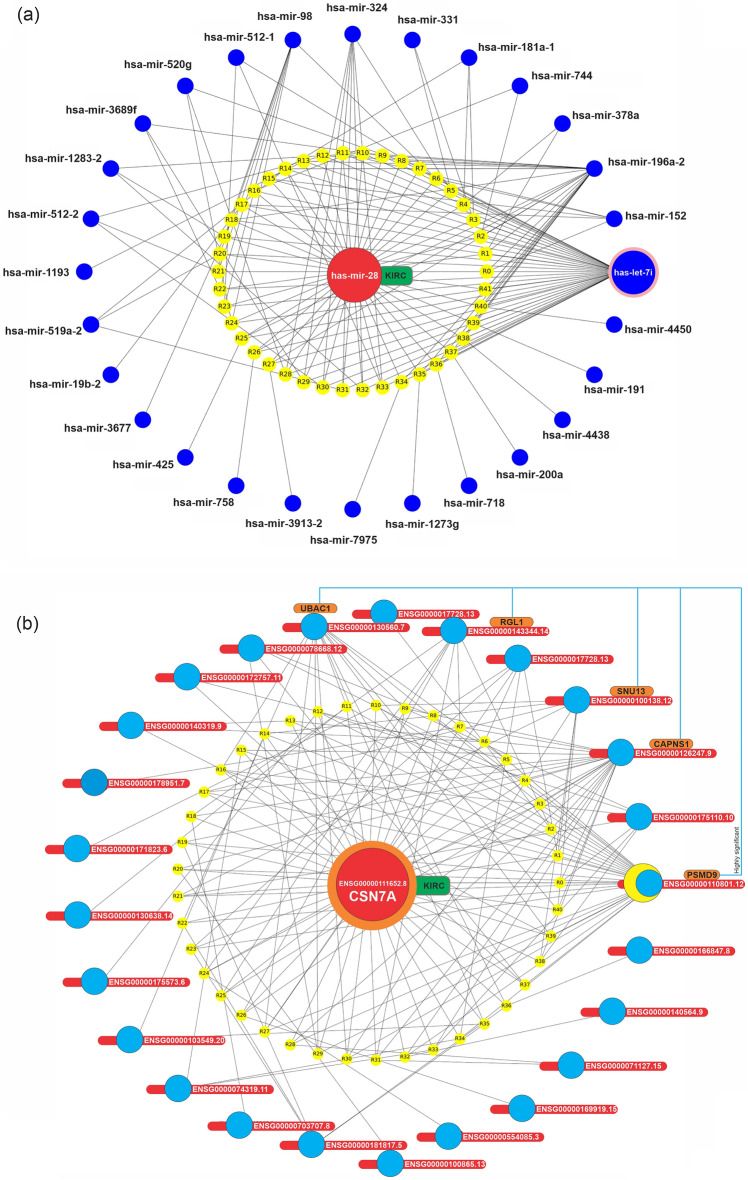
The relationship and specification of the miRNAs and mRNAs are based on the KIRC association rules. Graph network of (**a**) has-miR-28 (with support > 1.66) related association rules, in which the identified miRNA, its rules, and miRNAs were presented, in orange, yellow, and blue colors, respectively. (**b**) Graph network of ENSG00000111652.8 related association rules (with support > 0.3 and lift > 1.4), in which ENSG00000111652.8, rules, mRNAs were presented, in red, yellow, and blue colors, respectively.

**Figure 8:**
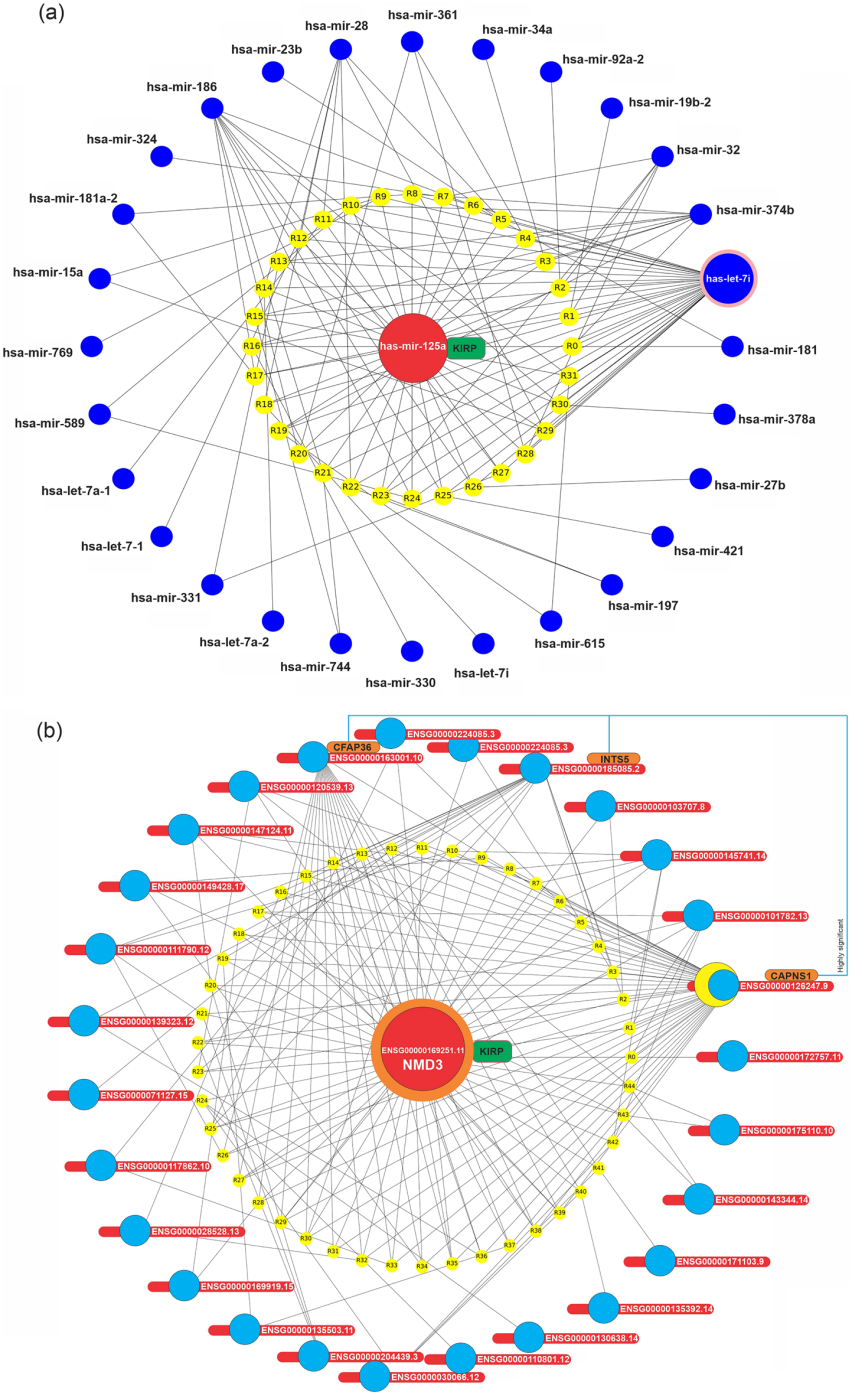
The relationship and specification of the miRNAs and mRNAs are based on the KIRP association rules. Graph network of (**a**) has-miR-125a (with support > 1.26) related association rules, in which the identified miRNA, its rules, and miRNAs were presented, in orange, yellow, and blue colors, respectively. (**b**) Graph network of ENSG00000169251.1 related association rules (with support > 0.2 and lift > 1.145), in which ENSG00000169251.1, rules, mRNAs were presented, in red, yellow, and blue colors, respectively.

CSN7A (ENSG00000111652.8) and NMD3 (ENSG00000169251.11) were the most frequent itemset with 5774 and 2306 repeat counts in KIRC and KIRP association rules, respectively. Moreover, miR-28 and miR-125a were the most frequent itemset with 2642 and 2591 repeat counts in KIRC and KIRP association rules, respectively. As a result, we decided to examine these miRNAs and mRNAs more closely using other association rules to investigate their relation with another feature. More in-depth coverage of these findings is available in the discussion section of the study. In this regard, we showed these relations based on association rules in the graph network (Figs. 7 and 8).

In Fig. 7a, it is obvious that hsa-miR-28, the most frequent miRNA in KIRC association rules, has a high dependency on hsa-let-7i, hsa-miR-196a-2, hsa-miR-324, hsa-miR-198, and hsa-miR-152, respectively. In Fig. 8a, it is clear that hsa-miR-125a, the most frequent miRNA in KIRP association rules, has a high dependency on hsa-miR-99b, hsa-miR-374b, hsa-miR-186, hsa-miR-28, and hsa-miR-32, respectively. In Fig. 7b, it is noticeable that CSN7A, the most frequent mRNA in KIRC association rules, has a high dependency on ENSG00000110801.12 (PSMD9), ENSG00000126247.9 (CAPNS1), ENSG00000130560.7 (UBAC1), ENSG00000100138.12 (SNU13), and ENSG00000143344.14 (RGL1: Ral guanine nucleotide dissociation stimulators like 1), respectively. In Fig. 8b, it is observable that ENSG00000169251.11 (NMD3), the most frequent miRNA in KIRP association rules, has a high dependency on ENSG00000126247.9 (CAPNS1), ENSG00000185085.2 INTS5 (integrator complex subunit 5), and ENSG00000163001.10 (CFAP36), respectively.

## Discussion

We carried out a comprehensive machine learning analysis of clinically significant patterns of the miRNAs and mRNAs within the RCC subtypes. A panel of 77 candidate mRNAs and a panel of 73 miRNAs could discriminate KIRC from KIRP with high accuracy. The association rule mining analysis could identify top mRNAs and miRNAs with the highest repeat counts, suggesting their possible pathological roles in each RCC subtype. The CSN7A and miR-28 along with the NMD3 and miR-125a were the most frequent itemsets in the KIRC and KIRP association rules, respectively. The roles of these mRNAs have not been studied before in the RCC. In the following sections, we present a brief discussion on the possible roles of these mRNAs and microRNAs in the pathogenesis of the KIRC and KIRP based on the published literature.

### Candidate RNAs in KIRC

The CSN7A, UBAC1, PSMD9, RNF40, and Capn4 were identified as candida mRNAs in the KIRC with the highest repeat counts by the association rule mining analysis. Machine learning approaches could detect novel targets in the field of the KIRC and bring the UPS (ubiquitin–proteasome system) and its components (CSN7A, UBAC1, PSMD9, and RNF40) into the focus of interest. The degradation of regulatory proteins by the UPS has an important function in controlling the cell cycle progression, DNA repair, response to extracellular stress, and signal transduction. The ubiquitin, proteasome, ubiquitinating enzymes (including ubiquitin-activating (E1), -conjugating (E2s), and -ligases (E3s) enzymes) along with deubiquitinating enzymes are the key components of the UPS system^36^. Most of the E3 ligases are cullin-RING ligases (CRLs) that determine the substrate-selectivity in response to specific stimuli either to degrade by the proteasome or modify their function. The COP9 signalosome (CSN), another component of the UPS, is a multi-subunit (CSN 1–8) metalloprotease complex^37^ that plays roles in gene expression, cell-cycle control, and DNA-damage response^38^, and increases the stability of some proteins including EGFR^39^. Furthermore, the CSN plays a role in controlling the activity of NF-κB, an inflammatory transcriptional regulator involved in cell survival, proliferation, and transformation^40,41^.

The VHL is the substrate recognition subunit of the E3 ubiquitin ligase complex that polyubiquitylates the hydroxylated targets under normoxia^42^. Under the loss of the VHL, the degradation of its specific targets, hypoxia inducible factors (HIF1α and HIF2α) does not occur, making a pseudohypoxia state. Both VHL deficiency and the accumulation of the HIF-1 promote the NF-κB activity that subsequently stimulates an NF-κB/PI3K/AKT/TGF-β/EGFR/IKK signaling cascade, resulting in the activation of proliferation and glycolytic pathways, a pro-angiogenic and apoptosis-resistant phenotype, and highly vascularized tumors^43,44^. It is reported that the CSN elevates the efficacy of the VHL-mediated HIF-1α recognizing, ubiquitination, and degradation^45^.

In our deep learning analysis, the CSN7A (COPS7A), 7a subunit of the CSN, was identified as the first top mRNA with the highest role in the KIRC. It is reported that in tumor tissues, the CSN7A level is decreased which may be associated with the oxidative phosphorylation pathway^46^ and the transcription-coupled nucleotide excision DNA repair^47^. Moreover, an overexpressed CSN7A could stimulate the IκBα deubiquitinylation and; consequently, suppressing the transcriptional activity of the NF-κB^48^. The mechanism by which the CSN7A can impact KIRC has not been defined; however, the CSN7A may function alike in the KIRC.

The UBAC1, the second identified mRNA in this study, is a subunit of the KPC complex, an E3 ubiquitin-protein ligase. The UBAC1 acts with the proteasome and ubiquitinated proteins such as the Nf-κB^49,50^. The UBAC1 also contributes to the inflammatory signal transduction pathways and affects cell proliferation and viability in keratinocytes^51^. The role of the UBAC1 in the KIRC needs to be clarified. The PSMD9 was 3^rd^ top KIRC-related mRNA found in our rule mining analysis. The PSMD9, as a part of the 26S proteasome, regulates protein degradation^52^. The PSMD9 is overexpressed in tumor tissues and associated with cell proliferation, hostile tumor outcome, and resistance to the therapy^53–55^. Since different tumor suppressors and oncogenes are controlled by the Ub- and proteosome-mediated degradation, the CSN7A, UBAC1, PSMD9, and RNF40 may play important roles in the pathogenesis of the KIRC. The CSN7A in the KIRC association rules has a high dependency on PSMD9, CAPNS1, and UBAC1, SNU13, respectively. Our study may open an innovative horizon to investigate the role of the CSN7A, UBAC1, PSMD9, and RNF40 in the pathogenesis of the KIRC.

microRNAs are critical managers of the development and progression of the RCC. They function as oncomirs or anti-oncomirs. miR-28, let-7i, miR-23b, miR-125a, miR-22 were top five identified miRNAs that were dysregulated in KIRC. For more details see Supplementary file, discussion part.

#### Candidate RNAs in KIRP

In the Rule mining analysis, the NMD3, ZNF41, CFAP36, FGFR1OP2, and RGL1 were identified as the top five mRNA with high association rules in the KIRP (≥ 2195).

The transcript patterns of the ribosomal proteins are tumor- and tissue-specific^56^ and their induced abnormal translation can provoke a malignant phenotype independent of chromatin remodeling and the deregulation of the transcriptional process^57^. The 60S ribosomal export protein NMD3 (NMD3) was the first identified mRNA involved in the pathogenesis of the KIRP in our study. The NMD3 is a nuclear adaptor protein that transports the 60S subunit into the cytoplasm via the nuclear pore complex and participates in the cytoplasmic maturation of 60S particles^58,59^. In the cytoplasm, the release of the NMD3p from 60S subunits needs a GTPase and the ribosomal protein (Rpl10p). Any mutation in these proteins leads to cytoplasmic retention of the NMD3 on pre-60S subunits, blocking ribosome assembly and biogenesis^60,61^. The NMD3 also exerts a significant effect on RNA biosynthesis, mainly ribosomal RNA, and consequently may impact tumorigenesis^62^. A direct role of the NMD3 needs to be elucidated in the KIRP.

**Figure 9:**
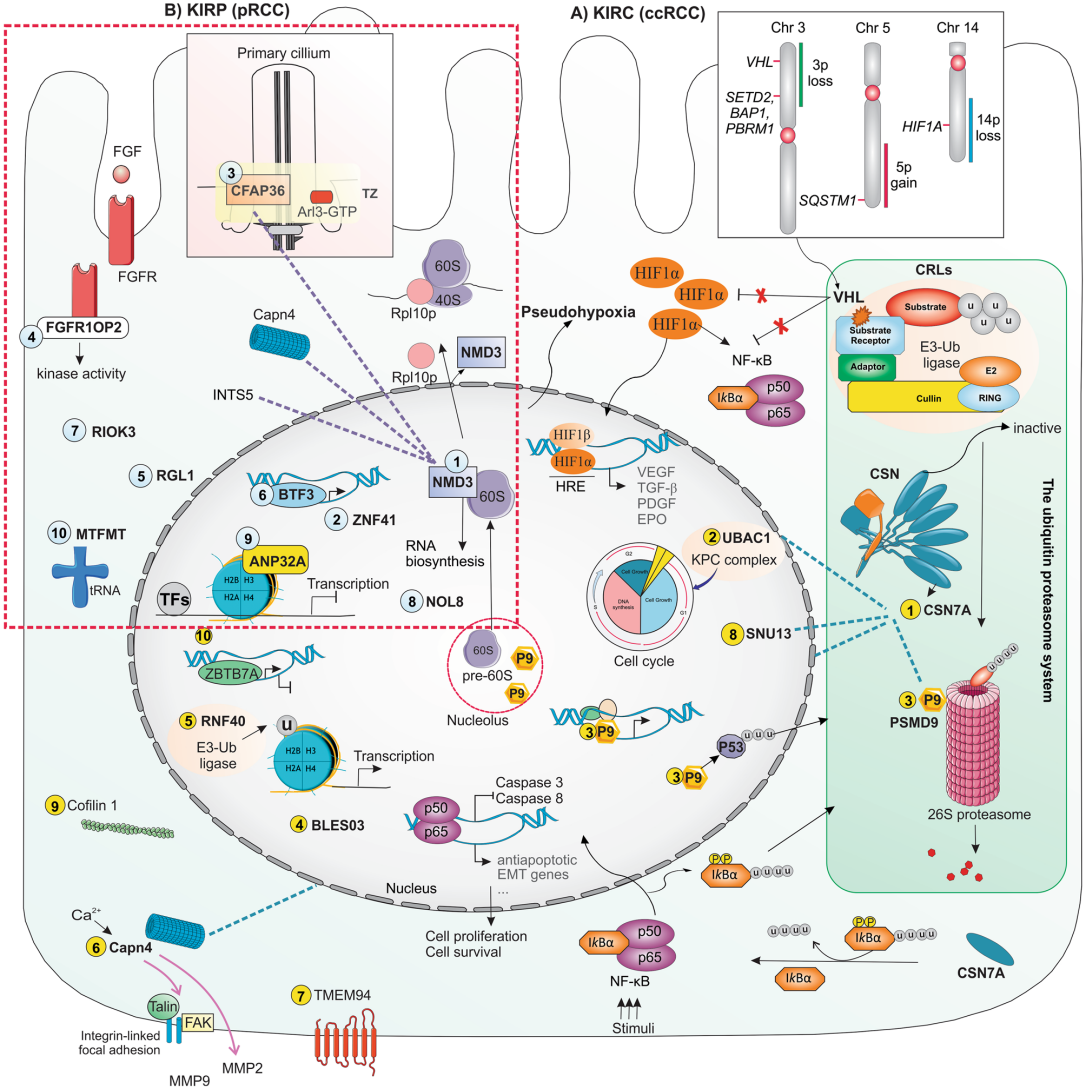
Possible roles of the deep learning-derived mRNAs and miRNAs in the pathogenesis of KIRC and KIRP (identified by deep learning methods). (**A**) The CSN7A, UBAC1, PSMD9, RNF40, Capn4, TMEMs, CFL1, CFL1, and ZBTB7A were identified as candida mRNAs (indicated by 1–10) in KIRC with the highest repeat counts by the Association Rule Mining analysis. The UPS is composed of Ub, proteasome, ubiquitinating (E1, E2s, and E3s), and deubiquitination enzymes. The VHL is the substrate recognition subunit of a CRL complex that polyubiquitylates its targets (HIF and NF-κB) to be degraded. In KIRC, the *VHL* gene is mutated or downregulated that result in the accumulation of these targets, making a pseudohypoxia state and highly vascularized tumors. The CSN7A, UBAC1, and PSMD9 are components of the UPS identified in this study. (1) CSN7A via IκBα deubiquitinylation can suppress the transcription activity of the NF-κB. (2) The UBAC1 is involved in ubiquitination and degradation of the cell cycle proteins. (3) the PSMD9 (P9) may be involved in the maintenance of integrity and morphology of nucleolus and impact cell survival and cell cycle regulation. The COPS7A in the KIRC association rules has a high dependency on PSMD9, Capn4, and UBAC1, SNU13, respectively (presented by dashed lines). (**B**) The NMD3, ZNF41, CFAP36, FGFR1OP2, RGL1, BTF3, RIOK3, NOL8, ANP32A, and MTFMT were identified as the top ten mRNA with high association rules in the KIRP. (1) The NMD3 is a nuclear adaptor protein that transports the ribosomal 60S subunit into the cytoplasm and participates in the cytoplasmic maturation of ribosomal 60S particles. It also affects RNA biosynthesis, mainly ribosomal RNA, and may impact tumorigenesis. (2) ZFP41 is a regulator of the transcription of different genes. (3) The CFAP36 is a binding partner of the Arl3, a small GTPase in the primary cilia. The primary cilia participate in the cilia formation, regulation of the cell cycle, and mitosis of cancer cells. The NMD3 in KIRP has a high dependency on CAPNS1, INTS5, and CFAP36, respectively (presented by dashed lines). For more details, please see the main text and Supplementary file, discussion part. *ANP32A* acidic nuclear phosphoprotein 32 family member A, *BLES03* basophilic leukemia-expressed protein, *BTF3* basic transcription factor 3, *CFAP36* Cilia and flagella-associated protein 36, *CAPN4* calpain small subunit 1, *CFL1* Cofilin1, *CRLs* E3 ligases are cullin-RING ligases, *CSN7A* COP9 signalosome subunit 7A, *FAK* focal adhesion kinase, *FGF* fibroblast growth factor, *FGFR1OP2* FGF receptor 1 oncogene partner 2, *HIF1* hypoxia inducible factors, *INTS5* integrator complex subunit 5, *MMP2/9* matrix metalloproteinase 2/9, *MTFMT* mitochondrial methionyl-tRNA formyltransferase, *NF-κB* nuclear factor kappa B, *NOL8* nucleolar protein 8, *PSMD9* proteasome 26S subunit, non-ATPase 9, *RGL1* Ral guanine nucleotide dissociation stimulators like 1, *RNF40* ring finger protein 40, *RIOK3* RIO kinase 3, *SNU13* small nuclear ribonucleoprotein 13, *TMEM94* transmembrane protein 94, *TZ* transition zone, *Ub* ubiquitin, *UBAC1* UBA domain containing 1, *UPS* ubiquitin–proteasome system, *ZBTB7A* zinc finger and BTB domain-containing 7A, *ZFP41* Zinc finger protein 41.

Respectively, miR-125a, miR-23b, miR-210, miR-99b, and miR-101-2 were the top five identified miRNAs dysregulated in KIRP. The possible pathological roles of the candidate mRNAs and miRNAs identified in this study are presented in Fig. 9 and explained in the Supplementary file in more detail. Although much remains to elucidate the KIRP mechanism, the roles of the NMD3, ZNF41, CFAP36, FGFR1OP2, and RGL1 are of considerable interest. The NMD3 in KIRP has a high dependency on CAPNS1, INTS5, and CFAP36, respectively.

The molecular features presented in this study will offer new insights into the underlying mechanisms that are responsible for the initiation and progression of KIRC and KIRP. Moreover, the ability to diagnose KIRC and KIRP with a high certainty level will help pathologists accuratelydifferentiate the most common subtypes of RCC. In this way, appropriate clinical decision-making strategies can be obtained. Furthermore, the ability of artificial intelligence for accurate differentiation of RCC may reduce unnecessary intervention rates. Our attempts to develop molecular discriminating patterns had some limitations. First of all, we did not evaluate the molecular mechanism of the candidate RNAs in the RCC models. In vitro and in vivo studies are needed to be performed to achieve this goal. Second, we did not validate the identified RNAs in clinical samples. Future studies would address this issue in large-sample-sized studies. We believe that adding other features from mutations, polymorphisms, alterations in copy number, and DNA methylation platforms would effectively tackle the discriminating problem of RCC subtypes and improve their early detection.

## Conclusion

In this paper, deep learning-driven biomarkers were presented for discriminating common subtypes of RCC. Panels of 77 mRNAs and 73 miRNAs could discriminate the KIRC, KIRP, and KICH subtypes from each other with high accuracy. The CSN7A and miR-28 along with the NMD3 and miR-125a were the most frequent itemsets in the KIRC and KIRP association rules, respectively. Due to a frequent mutation in protein-coding regions and an elevated burden of unfolded proteins; an elevated protein turnover was necessary for those speedily dividing cancer cells. Hence, the inhibition of the UPS components appeared to be a hopeful strategy for KIRC therapy. The identified mRNAs and microRNAs in this study can regulate signal transduction, cell cycle machinery, and apoptosis and all are relevant contributors to carcinogenesis and cancer progression. Therefore, they may provide further insight into the pathogenesis, diagnosis, prognosis, and molecular-targeted therapy in RCC subtypes (Fig. 9).

## Supplementary Information

Supplementary Information.

## Data Availability

The data obtained from the artificial intelligence approaches will be available from the corresponding authors upon request.
