# Supplementary material for: Panels of mRNAs and miRNAs for decoding molecular mechanisms of Renal Cell Carcinoma (RCC) subtypes utilizing Artificial Intelligence approaches
Source: Sci Rep. 2022 Sep 30;12:16393. doi: 10.1038/s41598-022-20783-7 (PMC9525704; doi:10.1038/s41598-022-20783-7)
Supplement: Supplementary file 1 — Supplementary Information. [file 41598_2022_20783_MOESM1_ESM.pdf]

Supplementary

Panels of mRNAs and miRNAs for decoding molecular mechanisms of Renal Cell Carcinoma (RCC) subtypes utilizing Artificial Intelligence approaches

Seyed Mahdi Hosseiniyan Khatibi, PhD[1, 2, 3], Mohammadreza Ardalan, MD[2], Mohammad Teshnehlab, PhD[4], Sepideh Zununi Vahed, Ph.D.[2, *], Saeed Pirmoradi, Ph.D. [1, 3, *]

Affiliations/institutions

[1]Clinical Research Development Unit of Tabriz Valiasr Hospital, Tabriz University of Medical Sciences, Tabriz, Iran

[2]Kidney Research Center, Tabriz University of Medical Sciences, Tabriz, Iran

[3]Rahat Breath and Sleep Research Center, Tabriz University of Medical Science, Tabriz, Iran

[4]Department of Electric and Computer Engineering, K.N. Toosi University of Technology, Tehran, Iran

***Corresponding authors:**
Sepideh Zununi Vahed
Kidney Research Center, Tabriz University of Medical Sciences, Tabriz, Iran
Daneshgah Street, Postal code 51665118, Tabriz, Iran.
Email: sepide.zununi@gmail.com

Saeed Pirmoradi
Clinical Research Development Unit of Tabriz Valiasr Hospital, Tabriz University of Medical Science, Tabriz, Iran
Valiasr Hospital, Zaferaniyeh, Tabriz, Iran.
Email: said.pirmoradi@gmail.com

Supplementary Methods

Classification

In this step, we applied a classifier to evaluate the candidate features selected in the previous step. High accuracy (or any user-defined measure) of classification demonstrates feature selection method was succeeded in choosing the relevant attributes. Otherwise feature selection method cannot identify relevant features.

Classification is defined as a process of predicting the class of given data points using mathematical methods. It is a task that needs to use machine learning algorithms, in which the machine learns how to assign a class label to samples from the problem field. Classes are sometimes called targets, labels, or categories. The classification model (classifier) learns to do mapping function (f) from features space (X) to discrete output variables (y) approximately. In this regard, the classifier applies input training data to predict the likelihood or probability in the data with predetermined categories. In machine learning, classification algorithms leverage a wide range of methods to classify datasets into correct categories.

In recent years, deep learning has been a new trend in the machine learning area, so it has succeeded in many applications with different domains [1]. Also, models based on deep learning have been widely applied in the health informatics field [2], such as translation bioinformatics, medical imaging, pervasive sensing for health, and medical informatics [3]. In this work, we employed a self-organizing deep auto-encoder model to classify data based on candidate features. A self-organizing deep auto-encoder is a specific type of deep auto-encoder that can determine its structure automatically, including the number of neurons and layers [4]. Description of self-organizing deep auto-encoder is available in more detail in First, the training process of the deep

model and the model selection was performed by training and validation data, respectively. Next, the performance of classification was estimated by employing test data.

Association Rule Mining

Table 1: Pseudocode of frequent itemset generation step in FP-Growth algorithm [5]

**Algorithm:** Frequent itemset generation in FP-Growth algorithm
**Input:** A database DB, represented by FP-tree constructed, and a minimum support threshold ξ.
**output:** The complete set of frequent patterns
**Method:** FP-growth (Tree, α)
(1) **if** Tree contains a single prefix path, **then**: // Mining single prefix-path FP-tree
(2) let P be the single prefix-path part of Tree;
(3) let Q be the multipath part with the top branching node replaced by a null root;
(4) **for** each combination (denoted as β) of the nodes in the path P **do**:
(5) generate pattern $\beta \cup \alpha$ with support = minimum support of nodes in β;
(6) let freq_pattern_set(P) be the set of patterns so generated;
(7) **end for**
(8) **else** let Q be Tree:
(9) **for** each item ai in Q **do**: // Mining multipath FP-tree
(10) generate pattern $\beta = a_i \cup \alpha$ with support = a_i.support;
(11) construct β's conditional pattern-base and then β's conditional FP-tree Treeβ;
(12) **if** Treeβ = ∅ **then**:
(13) call FP-growth (Treeβ, β);
(14) let freq_pattern_set(Q) be the set of patterns so generated;
(15) **end if**
(16) **end for**
(17) **end if**
(18) return (freq_pattern_set(P) ∪ freq_pattern_set(Q) ∪ (freq_pattern_set(P) × freq_pattern_set(Q)))

Table 2: Pseudocode of rules generation step in FP-Growth algorithm [6]

**Algorithm:** Rules generation in FP-Growth algorithm
**Method:**
(1) **For** each frequent itemset k-itemset F_k, k≥2 **do:**
(2) $H_1 = \{i \mid i \in F_k\}$; // 1-item consequents of the rule.
(3) call rules_generator (F_k, H_1);
(4) **End for**
Procedure rules_generator (F_k, H_m)
(1) $k = \|F_k\|$; // size of frequent itemset

$m = |H_m|$; // size of rule consequent

if k>m+1 **then:**

  $H_m = m + 1 - item\ consequents\ generated\ from\ H_m$;

  For each $h_{m+1} \in H_{m+1}$ **do:**

    $Confidance = \frac{Support(F_k)}{Support(F_k - H_{m+1})}$;

    if Confidence ≥ min_confidence **then:**

      output: the rule $(F_k - h_{m+1}) \rightarrow h_{m+1}$;

    Else:

      delete h_{m+1} from H_{m+1};

    End if

  End for

call ap-genrules(F_k, H_{m+1})

End if

Supplementary Tables

Supplementary Table 1. The list of 77 candidate mRNAs, was obtained from the feature selection step.

No.	mRNA ID	No.	mRNA ID	No.	mRNA ID
1	ENSG00000135392.14	27	ENSG00000078668.12	53	ENSG00000163001.10
2	ENSG00000183155.4	28	ENSG00000148606.11	54	ENSG00000138867.15
3	ENSG00000275066.3	29	ENSG00000224085.3	55	ENSG00000169919.15
4	ENSG00000088038.16	30	ENSG00000071127.15	56	ENSG00000149428.17
5	ENSG00000105085.9	31	ENSG00000074319.11	57	ENSG00000213341.9
6	ENSG00000147124.11	32	ENSG00000111790.12	58	ENSG00000116793.14
7	ENSG00000030066.12	33	ENSG00000171103.9	59	ENSG00000126247.9
8	ENSG00000204574.11	34	ENSG00000133142.16	60	ENSG00000140319.9
9	ENSG00000204439.3	35	ENSG00000171823.6	61	ENSG00000148337.18
10	ENSG00000143344.14	36	ENSG00000111325.15	62	ENSG00000114904.11
11	ENSG00000130638.14	37	ENSG00000137312.13	63	ENSG00000111652.8
12	ENSG00000174437.15	38	ENSG00000120438.10	64	ENSG00000166847.8
13	ENSG00000167925.14	39	ENSG00000100138.12	65	ENSG00000103549.20
14	ENSG00000028528.13	40	ENSG00000168734.12	66	ENSG00000117862.10
15	ENSG00000185085.2	41	ENSG00000177728.13	67	ENSG00000157869.13
16	ENSG00000172977.11	42	ENSG00000110801.12	68	ENSG00000213719.7
17	ENSG00000103707.8	43	ENSG00000145741.14	69	ENSG00000101782.13
18	ENSG00000175573.6	44	ENSG00000011260.12	70	ENSG00000152359.13
19	ENSG00000140350.14	45	ENSG00000120539.13	71	ENSG00000150316.10
20	ENSG00000172757.11	46	ENSG00000130560.7	72	ENSG00000198218.9
21	ENSG00000178951.7	47	ENSG00000105072.7	73	ENSG00000155463.11
22	ENSG00000100865.13	48	ENSG00000181817.5	74	ENSG00000183495.12
23	ENSG00000100330.14	49	ENSG00000177105.9	75	ENSG00000135503.11
24	ENSG00000198000.10	50	ENSG00000088888.16	76	ENSG00000108578.13
25	ENSG00000139323.12	51	ENSG00000140564.9	77	ENSG00000175110.10
26	ENSG00000169251.11	52	ENSG00000232024.2		

Supplementary Table 2. The list of 73 candidate miRNAs, which was obtained from feature selection step.

No.	miRNA ID	No.	miRNA ID	No.	miRNA ID
1	hsa-mir-99b	27	hsa-mir-1193	53	hsa-mir-512-2
2	hsa-let-7a-1	28	hsa-mir-28	54	hsa-mir-758
3	hsa-mir-330	29	hsa-mir-30a	55	hsa-mir-376a-1
4	hsa-mir-106b	30	hsa-mir-374b	56	hsa-mir-3689f
5	hsa-mir-196a-2	31	hsa-mir-454	57	hsa-mir-3913-2
6	hsa-mir-210	32	hsa-mir-15a	58	hsa-mir-377
7	hsa-mir-186	33	hsa-mir-652	59	hsa-mir-487a
8	hsa-mir-1254-2	34	hsa-mir-27b	60	hsa-mir-23a
9	hsa-mir-181a-2	35	hsa-mir-4438	61	hsa-mir-19b-2
10	hsa-mir-197	36	hsa-mir-26b	62	hsa-mir-615
11	hsa-mir-191	37	hsa-mir-331	63	hsa-mir-125a
12	hsa-mir-200a	38	hsa-mir-7-1	64	hsa-mir-371b
13	hsa-mir-130b	39	hsa-mir-769	65	hsa-mir-101-2
14	hsa-mir-425	40	hsa-mir-324	66	hsa-mir-181a-1
15	hsa-mir-589	41	hsa-mir-421	67	hsa-mir-520g
16	hsa-mir-34a	42	hsa-mir-519a-2	68	hsa-mir-92a-2
17	hsa-mir-4450	43	hsa-mir-1283-2	69	hsa-mir-942
18	hsa-mir-361	44	hsa-mir-1273g	70	hsa-let-7a-2
19	hsa-mir-152	45	hsa-let-7i	71	hsa-mir-1306
20	hsa-mir-4708	46	hsa-mir-590	72	hsa-mir-3677
21	hsa-mir-329-2	47	hsa-mir-23b	73	hsa-mir-744
22	hsa-mir-379	48	hsa-mir-2355		
23	hsa-mir-939	49	hsa-mir-32		
24	hsa-mir-98	50	hsa-mir-22		
25	hsa-mir-378a	51	hsa-mir-718		
26	hsa-mir-7975	52	hsa-mir-512-1		

Supplementary Table 3. Candidate miRNAs and mRNAs based on sorted repeat count.

miRNA				mRNA			
KIRC		KIRP		KIRC		KIRP	
ID	Repeat Count	ID	Repeat Count	ID	Repeat Count	ID	Repeat Count
hsa-mir-28	2642	hsa-mir-125a	2591	ENSG00000111652.8	5774	ENSG00000169251.11	2306
hsa-let-7i	2450	hsa-mir-23b	2415	ENSG00000130560.7	5690	ENSG00000147124.11	2285
hsa-mir-23b	2446	hsa-mir-210	2321	ENSG00000110801.12	5504	ENSG00000163001.10	2234
hsa-mir-125a	2266	hsa-mir-99b	2285	ENSG00000175573.6	5070	ENSG00000111790.12	2200
hsa-mir-22	2242	hsa-mir-101-2	2250	ENSG00000103549.20	5035	ENSG00000143344.14	2195
hsa-let-7a-2	2136	hsa-let-7i	2248	ENSG00000126247.9	4955	ENSG00000145741.14	2123
hsa-let-7a-1	2088	hsa-mir-28	2201	ENSG00000177728.13	4786	ENSG00000101782.13	2054
hsa-mir-99b	2071	hsa-mir-186	2187	ENSG00000100138.12	4446	ENSG00000198000.10	2042
hsa-mir-27b	2018	hsa-let-7a-1	2173	ENSG00000172757.11	4306	ENSG00000140350.14	1936
hsa-mir-186	1904	hsa-let-7a-2	2172	ENSG00000178951.7	4237	ENSG00000103707.8	1862
hsa-mir-210	1677	hsa-mir-7-1	2170	ENSG00000175110.10	4200	ENSG00000120539.13	1859
hsa-mir-7-1	1421	hsa-mir-590	1830	ENSG00000074319.11	4198	ENSG00000224085.3	1826
hsa-mir-23a	1419	hsa-mir-30a	1236	ENSG00000181817.5	4089	ENSG00000157869.13	1802
hsa-mir-32	1261	hsa-mir-2355	1159	ENSG00000078668.12	4049	ENSG00000204439.3	1776
hsa-mir-34a	952	hsa-mir-330	903	ENSG00000166847.8	3945	ENSG00000120438.10	1755
hsa-mir-421	936	hsa-mir-1306	874	ENSG00000143344.14	3933	ENSG00000149428.17	1675
hsa-mir-200a	931	hsa-mir-197	867	ENSG00000071127.15	3924	ENSG00000169919.15	1673
hsa-mir-191	930	hsa-mir-26b	857	ENSG00000108578.13	3848	ENSG00000028528.13	1610
hsa-mir-1283-2	928	hsa-mir-374b	856	ENSG00000155463.11	3809	ENSG00000167925.14	1503
hsa-mir-519a-2	928	hsa-mir-196a-2	818	ENSG00000088888.16	3784	ENSG00000177728.13	1468
hsa-mir-520g	927	hsa-mir-331	816	ENSG00000232024.2	3776	ENSG00000177105.9	1457
hsa-mir-3689f	927	hsa-mir-615	815	ENSG00000030066.12	3694	ENSG00000071127.15	1455
hsa-mir-512-2	927	hsa-mir-15a	805	ENSG00000140319.9	3590	ENSG00000150316.10	1436
hsa-mir-718	927	hsa-mir-454	790	ENSG00000138867.15	3554	ENSG00000204574.11	1416
hsa-mir-589	927	hsa-mir-371b	785	ENSG00000140564.9	3484	ENSG00000117862.10	1365
hsa-mir-512-1	927	hsa-mir-98	778	ENSG00000100865.13	3434	ENSG00000133142.16	1348
hsa-mir-152	926	hsa-mir-32	769	ENSG00000135503.11	3411	ENSG00000198218.9	1313
hsa-mir-1254-2	926	hsa-mir-939	751	ENSG00000137312.13	3373	ENSG00000185085.2	1280
hsa-mir-4450	925	hsa-mir-3677	749	ENSG00000198000.10	3365	ENSG00000140319.9	1279
hsa-mir-3913-2	925	hsa-mir-942	739	ENSG00000171823.6	3341	ENSG00000135503.11	1223
hsa-mir-7975	925	hsa-mir-487a	734	ENSG00000185085.2	3169	ENSG00000030066.12	1188
hsa-mir-652	924	hsa-mir-377	734	ENSG00000114904.11	3142	ENSG00000111652.8	1140
hsa-mir-4708	924	hsa-mir-376a-1	734	ENSG00000105072.7	3111	ENSG00000183155.4	1050
hsa-mir-130b	924	hsa-mir-379	734	ENSG00000103707.8	3086	ENSG00000100865.13	1039
hsa-mir-1193	923	hsa-mir-329-2	734	ENSG00000172977.11	3014	ENSG00000172977.11	1038
hsa-mir-1273g	923	hsa-mir-130b	731	ENSG00000169919.15	3009	ENSG00000126247.9	1036

Supplementary Figures

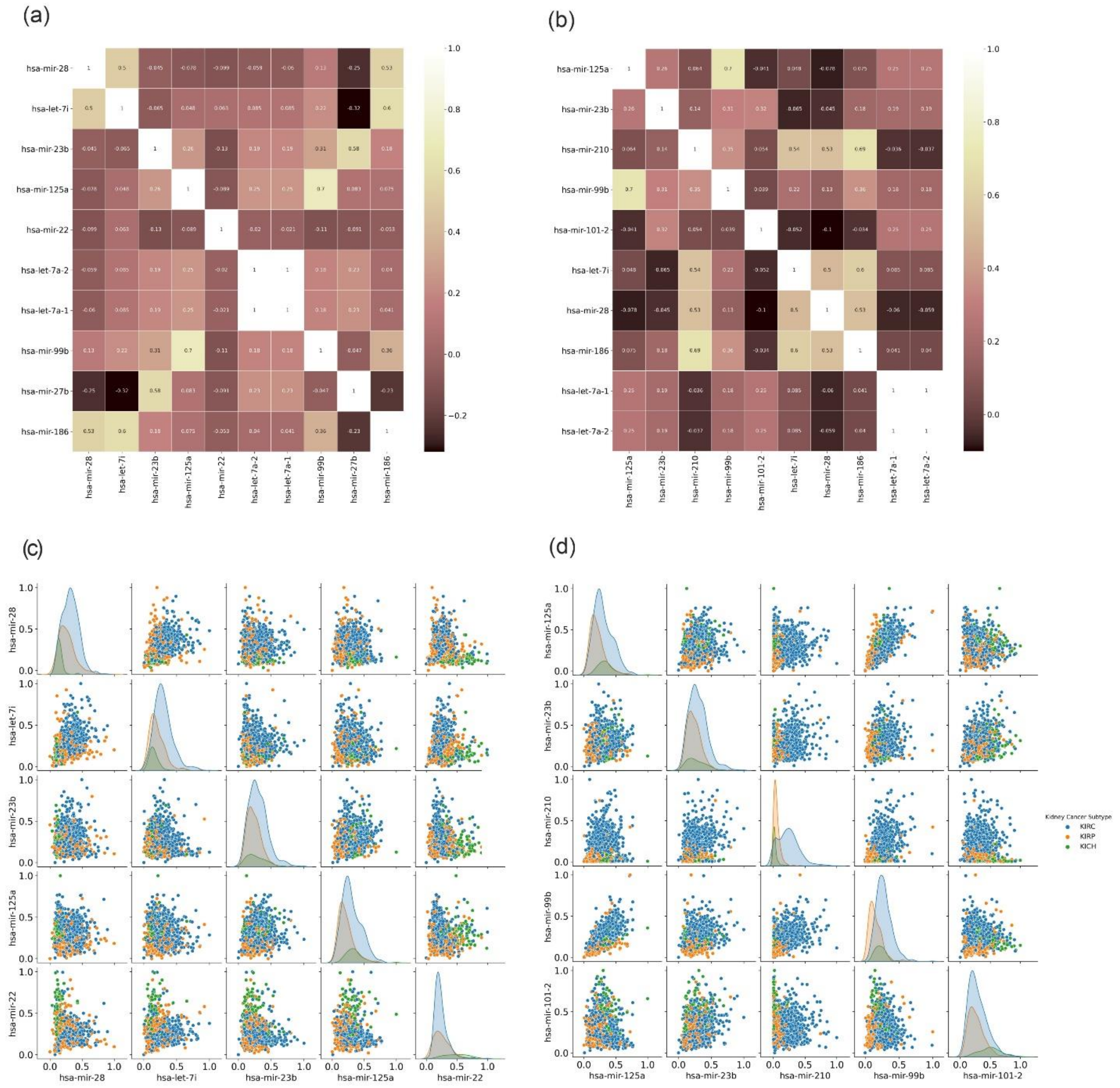

Supplementary Figure 1. The correlation heatmap plot is based on the Spearman correlation for ten top miRNAs of a) KIRC and b) KIRP rules. The pair plot of five top miRNAs of c) KIRC and d) KIRP rules.

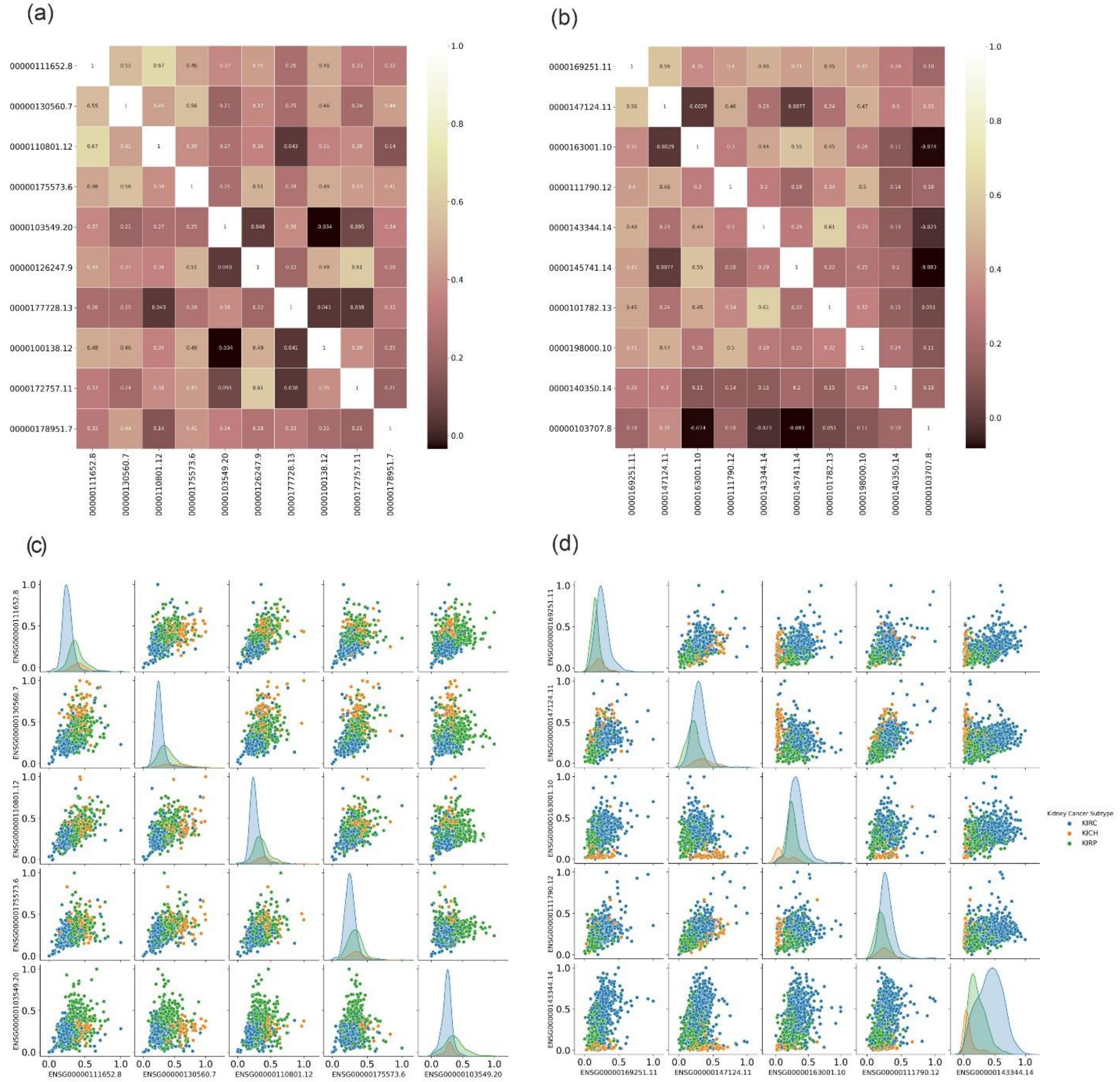

Supplementary Figure 2. The correlation heatmap plot is based on the Spearman correlation for ten top mRNAs of a) KIRC and b) KIRP rules. The pair plot of five top miRNAs of c) KIRC and d) KIRP rules.

Supplementary Discussion

Possible pathological roles of candidate transcripts in KIRC

As a transcriptional cofactor, the PSMD9 can control the translation, transcription, and receptor/ hormone activity through its interaction with the S14 (a ribosomal protein), CSH1 (a growth hormone), E12 (a transcription factor), and IL6 receptor[7]. It may participate in the activin A signaling cascade and growth regulation of cancer cells[8]. The interaction of the PSMD9 with the hnRNPA1 (heterogeneous nuclear ribonucleoprotein A1) results in the degradation of IkBa and the activation of NF-κB[9]. The PSMD9 also plays a role in the preservation of integrity and morphology of nucleolus and indirectly supports the p53 degradation by decreasing the free cytoplasmic ribosomal proteins to prevent the MDM2 E3 ligase activity. These functions overcome the anti-cancer drug-induced nucleolar stress and achieve a survival benefit for cells with the PSMD9. The absence of the PSMD9 (the nucleoplasm reorganization of free ribosomal proteins, p53 stabilization, and inhibition of MDM2 E3 ligase activity) indirectly can impact cell survival and cell cycle regulation[10].

The 4th identified top mRNA involved in the KIRC was basophilic leukemia-expressed protein (BLES03, *C11orf68).* The structure of this protein is similar to the eIF4E and its biological function is unknown. It might participate in a biochemical procedure that requires nucleic acids recognition[11]. An elevated gene expression of the BLES03 was seen in both primary tumors and cell lines of laryngeal squamous cell carcinoma due to the gain of copy number[12]. It is also reported that in metastatic prostate, liver, and breast cancer cell lines, the BLES03 is activated by hypomethylation[13]. It is indicated that the BLES03-like proteins can be novel eukaryotic phosphothreonine lyases that are involved in the dehydro amino acids biosynthesis[14]. The role of BLES03in the pathogenesis of KIRC needs to be investigated.

Epigenetic histone modifications, monoubiquitination of histone H2B (at lysine 120; H2Bub1), is connected with DNA damage response and active transcription. Human RING-finger protein 40 (RNF40) is an E3 ligase of H2B ubiquitination. Under DNA damage response, the interaction of p53 and RNF40/ RNF20/WAC complex regulates the transcription of genes[15]. The RNF40/20 complex also controls the p53-dependent mRNA splicing and gene transcription [16]. Moreover, this complex regulates DNA repair and chromatin stability and their aberrant expression causes replication stress and genomic instability, resulting in a dysregulated transcriptional program. By stimulating the NF-κB activity, the RNF40 has an essential role in the preservation of inflammatory signaling and tumorigenic features[17]. These data indicate that the RNF40 may be involved in the initial step of carcinogenesis[18]. However, it is not clear whether the RNF40 is a foe or friend in cancer. It may act as an oncogene in the liver, colorectal cancer[19], prostate cancer, and acute lymphoblastic leukemia[20] or a tumor-suppressor [21, 22]. The role of the RNF40 in the pathogenesis of the KIRC needs to be determined. Since different tumor suppressors and oncogenes are controlled by the Ub- and proteosome-mediated degradation, the CSN7A, UBAC1, PSMD9, and RNF40 may play important roles in the pathogenesis of the KIRC. The roles of the Capn4, TMEMs, and CFL1 are previously reported in the KIRC[23-25].

Possible pathological roles of candidate transcripts in KIRP

Zinc finger proteins (ZFPs) are transcription factors that through binding to the RNA or DNA play significant roles in the regulation of transcription, DNA repair, cell migration, degradation of Ub-protein, and signal transduction[26]. They are implicated in the progression of cancer by controlling the transcription of genes involved in apoptosis, migration, proliferation, and invasion[27]. They have crucial functions in the initiation and development of cancers especially

the RCC[28-30]. The ZNF41 is the second top mRNA identified in our deep-learning analysis. It is located on the X chromosome[31] and is associated with transcription regulation. The ZFP41 is identified as a cancer-related protein in malignant melanoma tissue[32]. In liver cancer, the ZFP41 gene is silenced via the hypermethylation of DNA[33]. The contribution of the ZNF41 in the pathogenesis of the KIRP may be associated with modifying cancer signaling pathways and its functions need to be studied.

Cilia and flagella-associated protein 36 (CFAP36 or CCDC104) was the third top identified mRNA in the KIRP. The CFAP36 is a binding partner of the Arl3, a small GTPase in the primary cilia[34]. The primary cilia, an antenna-like structure on the cells, is involved in the regulation of the cell cycle and organ homeostasis. The CFAP36 may also participate in cellular cilia formation and mitosis of cancer cells[35-37]. In the renal epithelium, the formation, function, and preservation of primary cilia are affected by the pVHL[38]. The loss of cilia is a common event underlying tumorigenesis in several subtypes of the RCC especially the KIRC, suggesting the role of nonciliated cells in cancer development[39]. In the KIRP with functioning pVHL, high frequencies of cilia were seen, indicating that this subtype can develop without disassembling its primary cilia. Evidence has unveiled reciprocal relations between the NRF2 and primary cilia. By inducing autophagy, primary cilia downregulate the NRF2 activity. On the other hand, the NRF2 controls genes complicated in Hedgehog (Hh) signaling and ciliogenesis transcriptionally, tumorigenesis, and stem cell function; however, the NRF2 may also impact these processes negatively[40, 41].

The fibroblast growth factor (FGF) and its receptor (FGFR) are critical factors in the transformation and tumorigenicity of the RCC [42, 43]. The FGFR pathway is complicated in driving tumor angiogenesis independent of the VEGF (vascular endothelial growth factor) to escape VEGF-targeted therapies[44]. The FGFR1 oncogene partner 2 (FGFR1OP2) was the 4th

identified mRNA in our deep-learning analysis. Fusion of the FGFR1OP2 to the FGFR1 gene happens in the myeloproliferative syndrome. The chimeric protein may display constitutive kinase activity that phosphorylates key signaling proteins involved in cancer cell viability and growth.

Ral guanine nucleotide dissociation stimulators like 1 (RGL1), basic transcription factor 3 (BTF3), RIO kinase 3, nucleolar protein 8 (NOL8), acidic nuclear phosphoprotein 32 family member A (ANP32A), and mitochondrial methionyl-tRNA formyltransferase (MTFMT) were other top 10 identified mRNAs with high roles in the pathogenesis of the KIRP. The functional roles of these factors need to be clarified.

Possible pathological roles of candidate microRNAs in the KIRC and KIRP

miR-28 presents contradictory roles in the RCC. In the context of the KIRC, a direct association was found between the elevated level of the miR-28-5p and the aneuploidy state that is induced by VHL depletion or pVHL loss of function. In VHL-associated cancers, upregulated miR-28-5p stimulates chromosomal instability by preventing the translation of the Mad2 [45]. On the other hand, a decreased level of the miR-28-5p was observed in different renal carcinoma cell lines and RCC tumor samples[46, 47]. As a tumor suppressor, the miR-28-5p inhibits proliferation and migration of the RCC cells by downregulating the RAP1B, a GTPase, and affecting the activation of the Erk1/2 and p38 MAP kinases[46].

Upregulated levels of the let-7i-5p were connected with high progression or grade/stage of the KIRC[48] and poor prognosis[49]. The let-7i-5p can stimulate the KIRC cells' proliferation, invasion, and migration, by downregulating its target hyaluronan-binding protein 4 (HABP4 or Ki-1/57), a tumor suppressor gene[50]. Moreover, a dysregulated level of the let-7i was observed in plasma exosomes of patients with metastatic renal cancer[51].

The miR-23b-3p acts as an oncomiR by targeting PTEN[52] and proline oxidase[53] in the RCC. By generating ROS (reactive oxygen species) and reducing HIF signaling, mitochondrial proline oxidase induces apoptosis and prevents cell proliferation[53]. In contrast to this study, a decreased expression level of the miR-23b was seen in the KIRC tissue that was associated with pathological stage/grade and a high risk of cancer progression[54]. It is indicated that the cytokine-cytokine receptor interaction pathway was the major pathway controlled by the miR-23b in the RCC. Colony-stimulating factor 1, epidermal growth factor receptor, MET proto-oncogene, Il-21 receptor, and Chemokine (C-X-C motif) ligand 11 were the main targets of the miR-23b in this pathway[55]. Additionally, an association was reported between prolonged response to sunitinib and elevated levels of the miR-23b in patients with metastatic RCC[56].

The miR-125a is a tumor suppressor that regulates hyaluronic acid synthase 1 and cellular proliferation, apoptosis, and migration by targeting the STAT3 in the RCC [57, 58]. The miR-125a-3p expression is activated by the PinX1, a tumor suppressor. The VEGF is a direct target of the mir-125a-3p. miR-125a-3p/VEGF signal pathway is involved in renal cancer angiogenesis[59]. The miR-22 acts differently in tumor progress and is down-regulated in cell lines, serum, and tissues of patients with the RCC [60-62]. On the other hand, as a master oncomir, the miR-22 controls the expression of genes related to survival in the KIRC by stimulating cellular invasion[63]. Moreover, the miR-22 impacts the proliferation and migration of KIRC cells by targeting SIRT1, PTEN, CREB1, and PI3K/AKT pathways[62, 64-66].

Plenty of articles have studied the impact of the miR-210 in the RCC indicating that miR-210 functions as an oncomir in the RCC[67, 68], especially in the KIRP[69]. The results of two meta-analyses indicated that the miR-210 may be a diagnostic biomarker in the RCC[54, 70]. As an onco-suppressor, the miR-99b represses mTOR and IGF1R expression to down-regulate

mTOR/AKT/IGF1Rsignaling[71]. The low expression level of the miR-99b-5p might correlate with tumor progression in the KIRC patients treated with tyrosine kinase inhibitor[72].

The miR-101 is induced by hypoxia, promoting glycolysis by targeting TIGAR (TP53‑induced glycolysis and apoptosis regulator) in the KIRC[73]. As a tumor suppressor, the miR-101-3p inhibits cell proliferation, invasion, and migration of the RCC by targeting the EZH2S, *DONSON* [74], and oncogenic factors involved in the pathogenesis of the KIRC[75, 76]. Moreover, the miR-101 by targeting the *UHRF1* suppresses nucleotide excision repair and mismatch repair[77].

Collectively, due to a frequent mutation in protein-coding regions and an elevated burden of unfolded proteins, elevated protein turnover is necessary for those speedily dividing cancer cells; and observed up-regulated proteasome assembling proteins are an adopting mechanism to reach this utmost need. Hence, the inhibition of the UPS components appears to be a hopeful strategy for KIRC therapy. Our study may open an innovative horizon to investigate the role of the CSN7A, UBAC1, PSMD9, and RNF40 in the pathogenesis of the KIRC. The COPS7A in the KIRC association rules has a high dependency on PSMD9, CAPNS1, and UBAC1, SNU13, respectively. Although much remains to elucidate the KIRP mechanism, the roles of the NMD3, ZNF41, CFAP36, FGFR1OP2, and RGL1 are of considerable interest. The NMD3 in KIRP has a high dependency on CAPNS1, INTS5 (integrator complex subunit 5), and CFAP36, respectively.

References

[1] S. Pouyanfar *et al.*, "A Survey on Deep Learning: Algorithms, Techniques, and Applications," vol. 51, no. 5, pp. 1-36, 2018, doi: 10.1145/3234150.

[2] R. Miotto, F. Wang, S. Wang, X. Jiang, and J. T. Dudley, "Deep learning for healthcare: review, opportunities and challenges," *Briefings in Bioinformatics,* vol. 19, no. 6, pp. 1236-1246, 2017, doi: 10.1093/bib/bbx044 %J Briefings in Bioinformatics.

[3] D. Ravì *et al.*, "Deep learning for health informatics," *IEEE journal of biomedical and health informatics,* vol. 21, no. 1, pp. 4-21, 2016.

[4] S. Pirmoradi, M. Teshnehlab, N. Zarghami, and A. Sharifi, "A Self-organizing Deep Auto-Encoder approach for Classification of Complex Diseases using SNP Genomics Data," *Applied Soft Computing*, p. 106718, 2020/09/12/ 2020, doi: https://doi.org/10.1016/j.asoc.2020.106718.
[5] J. Han, J. Pei, Y. Yin, and R. Mao, "Mining frequent patterns without candidate generation: A frequent-pattern tree approach," *Data mining and knowledge discovery*, vol. 8, no. 1, pp. 53-87, 2004.
[6] J. Xie, J. Wu, and Q. Qian, "Feature selection algorithm based on association rules mining method," in *2009 Eighth IEEE/ACIS International Conference on Computer and Information Science*, 2009: IEEE, pp. 357-362.
[7] N. Sangith *et al.*, "Discovery of novel interacting partners of PSMD9, a proteasomal chaperone: Role of an Atypical and versatile PDZ-domain motif interaction and identification of putative functional modules," (in eng), *FEBS open bio*, vol. 4, pp. 571-83, 2014, doi: 10.1016/j.fob.2014.05.005.
[8] C. Banz-Jansen, B. Münchow, K. Diedrich, and D. Finas, "Bridge-1 is expressed in human breast carcinomas: silencing of Bridge-1 decreases Smad2, Smad3 and Smad4 expression in MCF-7 cells, a human breast cancer cell line," (in eng), *Archives of gynecology and obstetrics*, vol. 284, no. 6, pp. 1543-9, Dec 2011, doi: 10.1007/s00404-011-1875-0.
[9] I. Sahu, N. Sangith, M. Ramteke, R. Gadre, and P. Venkatraman, "A novel role for the proteasomal chaperone PSMD9 and hnRNPA1 in enhancing IκBα degradation and NF-κB activation - functional relevance of predicted PDZ domain-motif interaction," (in eng), *The FEBS journal*, vol. 281, no. 11, pp. 2688-709, Jun 2014, doi: 10.1111/febs.12814.
[10] S. B. Ud Din Farooqee, J. Christie, and P. Venkatraman, "PSMD9 ribosomal protein network maintains nucleolar architecture and WT p53 levels," (in eng), *Biochemical and biophysical research communications*, vol. 563, pp. 105-112, Jul 23 2021, doi: 10.1016/j.bbrc.2021.05.004.
[11] E. Bitto, C. A. Bingman, H. Robinson, S. T. Allard, G. E. Wesenberg, and G. N. Phillips, Jr., "The structure at 2.5 A resolution of human basophilic leukemia-expressed protein BLES03," (in eng), *Acta crystallographica. Section F, Structural biology and crystallization communications*, vol. 61, no. Pt 9, pp. 812-7, Sep 1 2005, doi: 10.1107/s1744309105023845.
[12] A. K. Järvinen *et al.*, "Identification of target genes in laryngeal squamous cell carcinoma by high-resolution copy number and gene expression microarray analyses," (in eng), *Oncogene*, vol. 25, no. 52, pp. 6997-7008, Nov 2 2006, doi: 10.1038/sj.onc.1209690.
[13] D. Cheishvili *et al.*, "A common promoter hypomethylation signature in invasive breast, liver and prostate cancer cell lines reveals novel targets involved in cancer invasiveness," (in eng), *Oncotarget*, vol. 6, no. 32, pp. 33253-68, Oct 20 2015, doi: 10.18632/oncotarget.5291.
[14] S. Khater and D. Mohanty, "Genome-wide search for eliminylating domains reveals novel function for BLES03-like proteins," (in eng), *Genome biology and evolution*, vol. 6, no. 8, pp. 2017-33, Jul 24 2014, doi: 10.1093/gbe/evu161.
[15] D. Meng, K. Guo, D. Zhang, C. Zhao, C. Sun, and F. Zhang, "Ring finger 20/ring finger 40/WW domain-containing adaptor with coiled-coil complex interacts with p53 to regulate gene transcription in DNA damage response," (in eng), *Oncology letters*, vol. 21, no. 6, p. 436, Jun 2021, doi: 10.3892/ol.2021.12697.
[16] C. Wu, Y. Cui, X. Liu, F. Zhang, L. Y. Lu, and X. Yu, "The RNF20/40 complex regulates p53-dependent gene transcription and mRNA splicing," (in eng), *Journal of molecular cell biology*, vol. 12, no. 2, pp. 113-124, Feb 20 2020, doi: 10.1093/jmcb/mjz045.
[17] R. L. Kosinsky *et al.*, "Loss of RNF40 Decreases NF-κB Activity in Colorectal Cancer Cells and Reduces Colitis Burden in Mice," (in eng), *Journal of Crohn's & colitis*, vol. 13, no. 3, pp. 362-373, Mar 26 2019, doi: 10.1093/ecco-jcc/jjy165.
[18] S. B. Chernikova *et al.*, "Deficiency in mammalian histone H2B ubiquitin ligase Bre1 (Rnf20/Rnf40) leads to replication stress and chromosomal instability," (in eng), *Cancer research*, vol. 72, no. 8, pp. 2111-9, Apr 15 2012, doi: 10.1158/0008-5472.Can-11-2209.

[19] D. Schneider *et al.*, "The E3 ubiquitin ligase RNF40 suppresses apoptosis in colorectal cancer cells," (in eng), *Clinical epigenetics,* vol. 11, no. 1, p. 98, Jul 2 2019, doi: 10.1186/s13148-019-0698-x.
[20] X. Zheng, K. Chen, X. Liu, Y. Pan, and H. Liu, "High RNF40 expression indicates poor prognosis of hepatocellular carcinoma," (in eng), *International journal of clinical and experimental pathology,* vol. 11, no. 5, pp. 2901-2906, 2018.
[21] F. Wegwitz *et al.*, "The histone H2B ubiquitin ligase RNF40 is required for HER2-driven mammary tumorigenesis," (in eng), *Cell death & disease,* vol. 11, no. 10, p. 873, Oct 17 2020, doi: 10.1038/s41419-020-03081-w.
[22] J. Fu, L. Liao, K. S. Balaji, C. Wei, J. Kim, and J. Peng, "Epigenetic modification and a role for the E3 ligase RNF40 in cancer development and metastasis," (in eng), *Oncogene,* vol. 40, no. 3, pp. 465-474, Jan 2021, doi: 10.1038/s41388-020-01556-w.
[23] Q. Zhuang *et al.*, "Capn4 contributes to tumor invasion and metastasis in clear cell renal cell carcinoma cells via modulating talin-focal adhesion kinase signaling pathway," (in eng), *Acta biochimica et biophysica Sinica,* vol. 50, no. 5, pp. 465-472, May 1 2018, doi: 10.1093/abbs/gmy031.
[24] Q. Zhuang *et al.*, "Overexpression of Capns1 Predicts Poor Prognosis and Correlates with Tumor Progression in Renal Cell Carcinoma," (in eng), *Urologia internationalis*, vol. 105, no. 7-8, pp. 697-704, 2021, doi: 10.1159/000511638.
[25] Q. Zhuang, X. Qian, Y. Cao, M. Fan, X. Xu, and X. He, "Capn4 mRNA level is correlated with tumour progression and clinical outcome in clear cell renal cell carcinoma," (in eng), *The Journal of international medical research,* vol. 42, no. 2, pp. 282-91, Apr 2014, doi: 10.1177/0300060513505524.
[26] M. Cassandri *et al.*, "Zinc-finger proteins in health and disease," (in eng), *Cell death discovery,* vol. 3, p. 17071, 2017, doi: 10.1038/cddiscovery.2017.71.
[27] J. Jen and Y. C. Wang, "Zinc finger proteins in cancer progression," (in eng), *Journal of biomedical science,* vol. 23, no. 1, p. 53, Jul 13 2016, doi: 10.1186/s12929-016-0269-9.
[28] Y. J. Kim *et al.*, "ZNF492 and GPR149 methylation patterns as prognostic markers for clear cell renal cell carcinoma: Array-based DNA methylation profiling," (in eng), *Oncol Rep,* vol. 42, no. 1, pp. 453-460, Jul 2019, doi: 10.3892/or.2019.7151.
[29] G. Li *et al.*, "Overexpression of antisense long non-coding RNA ZNF710-AS1-202 promotes cell proliferation and inhibits apoptosis of clear cell renal cell carcinoma via regulation of ZNF710 expression," (in eng), *Molecular medicine reports,* vol. 21, no. 6, pp. 2502-2512, Jun 2020, doi: 10.3892/mmr.2020.11032.
[30] G. S. Dalgin, D. T. Holloway, L. S. Liou, and C. DeLisi, "Identification and characterization of renal cell carcinoma gene markers," (in eng), *Cancer informatics,* vol. 3, pp. 65-92, Feb 9 2007.
[31] M. Rosati, A. Franzé, M. R. Matarazzo, and G. Grimaldi, "Coding region intron/exon organization, alternative splicing, and X-chromosome inactivation of the KRAB/FPB-domain-containing human zinc finger gene ZNF41," (in eng), *Cytogenetics and cell genetics,* vol. 85, no. 3-4, pp. 291-6, 1999, doi: 10.1159/000015315.
[32] A. Sanchez *et al.*, "Novel functional proteins coded by the human genome discovered in metastases of melanoma patients," (in eng), *Cell biology and toxicology,* vol. 36, no. 3, pp. 261-272, Jun 2020, doi: 10.1007/s10565-019-09494-4.
[33] E. Arai, T. Yotani, and Y. Kanai, "DNA and Histone Methylation in Liver Cancer," in *DNA and Histone Methylation as Cancer Targets*: Springer, 2017, pp. 437-460.
[34] L. Powell, Y. H. Samarakoon, S. Ismail, and J. A. Sayer, "ARL3, a small GTPase with a functionally conserved role in primary cilia and immune synapses," (in eng), *Small GTPases,* vol. 12, no. 3, pp. 167-176, May 2021, doi: 10.1080/21541248.2019.1703466.
[35] T. W. Eichler, C. Totland, M. Haugen, and C. A. Vedeler, "CCDC104 Antibodies and Mitosis of Cancer Cells," (in eng), *Scandinavian journal of immunology,* vol. 87, no. 2, pp. 109-110, Feb 2018, doi: 10.1111/sji.12634.

[36] M. Lokaj *et al.*, "The Interaction of CCDC104/BARTL1 with Arl3 and Implications for Ciliary Function," (in eng), *Structure (London, England : 1993),* vol. 23, no. 11, pp. 2122-32, Nov 3 2015, doi: 10.1016/j.str.2015.08.016.
[37] C. Totland, G. Bredholt, M. Haugen, B. I. Haukanes, and C. A. Vedeler, "Antibody to CCDC104 is associated with a paraneoplastic antibody to CDR2 (anti-Yo)," (in eng), *Cancer immunology, immunotherapy : CII,* vol. 59, no. 2, pp. 231-7, Feb 2010, doi: 10.1007/s00262-009-0742-3.
[38] M. A. Esteban, S. K. Harten, M. G. Tran, and P. H. Maxwell, "Formation of primary cilia in the renal epithelium is regulated by the von Hippel-Lindau tumor suppressor protein," (in eng), *Journal of the American Society of Nephrology : JASN,* vol. 17, no. 7, pp. 1801-6, Jul 2006, doi: 10.1681/asn.2006020181.
[39] S. G. Basten *et al.*, "Reduced cilia frequencies in human renal cell carcinomas versus neighboring parenchymal tissue," (in eng), *Cilia,* vol. 2, no. 1, p. 2, Jan 31 2013, doi: 10.1186/2046-2530-2-2.
[40] A. Martin-Hurtado, I. Lastres-Becker, A. Cuadrado, and F. R. Garcia-Gonzalo, "NRF2 and Primary Cilia: An Emerging Partnership," (in eng), *Antioxidants (Basel, Switzerland),* vol. 9, no. 6, Jun 2 2020, doi: 10.3390/antiox9060475.
[41] P. Liu, M. Dodson, D. Fang, E. Chapman, and D. D. Zhang, "NRF2 negatively regulates primary ciliogenesis and hedgehog signaling," (in eng), *PLoS biology,* vol. 18, no. 2, p. e3000620, Feb 2020, doi: 10.1371/journal.pbio.3000620.
[42] I. Tsimafeyeu, L. Demidov, E. Stepanova, N. Wynn, and H. Ta, "Overexpression of fibroblast growth factor receptors FGFR1 and FGFR2 in renal cell carcinoma," (in eng), *Scandinavian journal of urology and nephrology,* vol. 45, no. 3, pp. 190-5, Apr 2011, doi: 10.3109/00365599.2011.552436.
[43] M. Volkova *et al.*, "Immunochemical expression of fibroblast growth factor and its receptors in primary tumor cells of renal cell carcinoma," (in eng), *American journal of clinical and experimental urology,* vol. 9, no. 1, pp. 65-72, 2021.
[44] F. Massari *et al.*, "Targeting fibroblast growth factor receptor (FGFR) pathway in renal cell carcinoma," (in eng), *Expert review of anticancer therapy,* vol. 15, no. 12, pp. 1367-9, 2015, doi: 10.1586/14737140.2015.1110488.
[45] M. P. Hell, C. R. Thoma, N. Fankhauser, Y. Christinat, T. C. Weber, and W. Krek, "miR-28-5p promotes chromosomal instability in VHL-associated cancers by inhibiting Mad2 translation," (in eng), *Cancer research,* vol. 74, no. 9, pp. 2432-43, May 1 2014, doi: 10.1158/0008-5472.Can-13-2041.
[46] C. Wang *et al.*, "miR-28-5p acts as a tumor suppressor in renal cell carcinoma for multiple antitumor effects by targeting RAP1B," (in eng), *Oncotarget,* vol. 7, no. 45, pp. 73888-73902, Nov 8 2016, doi: 10.18632/oncotarget.12516.
[47] C. Wang *et al.*, "A panel of five serum miRNAs as a potential diagnostic tool for early-stage renal cell carcinoma," (in eng), *Sci Rep,* vol. 5, p. 7610, Jan 5 2015, doi: 10.1038/srep07610.
[48] B. Gowrishankar *et al.*, "MicroRNA expression signatures of stage, grade, and progression in clear cell RCC," (in eng), *Cancer biology & therapy,* vol. 15, no. 3, pp. 329-41, Mar 1 2014, doi: 10.4161/cbt.27314.
[49] A. H. Girgis *et al.*, "Multilevel whole-genome analysis reveals candidate biomarkers in clear cell renal cell carcinoma," (in eng), *Cancer research,* vol. 72, no. 20, pp. 5273-84, Oct 15 2012, doi: 10.1158/0008-5472.Can-12-0656.
[50] Y. Liu, X. Hu, L. Hu, C. Xu, and X. Liang, "Let-7i-5p enhances cell proliferation, migration and invasion of ccRCC by targeting HABP4," (in eng), *BMC urology,* vol. 21, no. 1, p. 49, Mar 28 2021, doi: 10.1186/s12894-021-00820-9.
[51] M. Du *et al.*, "Plasma exosomal miRNAs-based prognosis in metastatic kidney cancer," (in eng), *Oncotarget,* vol. 8, no. 38, pp. 63703-63714, Sep 8 2017, doi: 10.18632/oncotarget.19476.
[52] M. S. Zaman *et al.*, "Inhibition of PTEN gene expression by oncogenic miR-23b-3p in renal cancer," (in eng), *PloS one,* vol. 7, no. 11, p. e50203, 2012, doi: 10.1371/journal.pone.0050203.

[53] W. Liu *et al.*, "miR-23b targets proline oxidase, a novel tumor suppressor protein in renal cancer," (in eng), *Oncogene,* vol. 29, no. 35, pp. 4914-24, Sep 2 2010, doi: 10.1038/onc.2010.237.
[54] L. Gu *et al.*, "MicroRNAs as prognostic molecular signatures in renal cell carcinoma: a systematic review and meta-analysis," (in eng), *Oncotarget,* vol. 6, no. 32, pp. 32545-60, Oct 20 2015, doi: 10.18632/oncotarget.5324.
[55] T. Ishihara *et al.*, "Expression of the tumor suppressive miRNA-23b/27b cluster is a good prognostic marker in clear cell renal cell carcinoma," (in eng), *J Urol,* vol. 192, no. 6, pp. 1822-30, Dec 2014, doi: 10.1016/j.juro.2014.07.001.
[56] J. Puente *et al.*, "Novel potential predictive markers of sunitinib outcomes in long-term responders versus primary refractory patients with metastatic clear-cell renal cell carcinoma," (in eng), *Oncotarget,* vol. 8, no. 18, pp. 30410-30421, May 2 2017, doi: 10.18632/oncotarget.16494.
[57] D. Chen *et al.*, "Identification of miR-125a-5p as a tumor suppressor of renal cell carcinoma, regulating cellular proliferation, migration and apoptosis," (in eng), *Molecular medicine reports,* vol. 11, no. 2, pp. 1278-83, Feb 2015, doi: 10.3892/mmr.2014.2848.
[58] M. Sun *et al.*, "MicroRNA-125a suppresses cell migration, invasion, and regulates hyaluronic acid synthase 1 expression by targeting signal transducers and activators of transcription 3 in renal cell carcinoma cells," (in eng), *Journal of cellular biochemistry,* vol. 120, no. 2, pp. 1894-1902, Feb 2019, doi: 10.1002/jcb.27503.
[59] P. Hou *et al.*, "PinX1 represses renal cancer angiogenesis via the mir-125a-3p/VEGF signaling pathway," (in eng), *Angiogenesis,* vol. 22, no. 4, pp. 507-519, Nov 2019, doi: 10.1007/s10456-019-09675-z.
[60] M. Li, Y. Sha, and X. Zhang, "MiR-22 functions as a biomarker and regulates cell proliferation, cycle, apoptosis, migration and invasion in renal cell carcinoma," (in eng), *International journal of clinical and experimental pathology,* vol. 10, no. 12, pp. 11425-11437, 2017.
[61] H. He *et al.*, "MicroRNA Expression Profiling in Clear Cell Renal Cell Carcinoma: Identification and Functional Validation of Key miRNAs," (in eng), *PloS one,* vol. 10, no. 5, p. e0125672, 2015, doi: 10.1371/journal.pone.0125672.
[62] S. Zhang, D. Zhang, C. Yi, Y. Wang, H. Wang, and J. Wang, "MicroRNA-22 functions as a tumor suppressor by targeting SIRT1 in renal cell carcinoma," (in eng), *Oncol Rep,* vol. 35, no. 1, pp. 559-67, Jan 2016, doi: 10.3892/or.2015.4333.
[63] X. Gong, H. Zhao, M. Saar, D. M. Peehl, and J. D. Brooks, "miR-22 Regulates Invasion, Gene Expression and Predicts Overall Survival in Patients with Clear Cell Renal Cell Carcinoma," (in eng), *Kidney cancer (Clifton, Va.),* vol. 3, no. 2, pp. 119-132, Aug 7 2019, doi: 10.3233/kca-190051.
[64] Z. Li, Z. Ma, and X. Xu, "Long non-coding RNA MALAT1 correlates with cell viability and mobility by targeting miR-22-3p in renal cell carcinoma via the PI3K/Akt pathway," (in eng), *Oncol Rep,* vol. 41, no. 2, pp. 1113-1121, Feb 2019, doi: 10.3892/or.2018.6853.
[65] W. Fan, J. Huang, H. Xiao, and Z. Liang, "MicroRNA-22 is downregulated in clear cell renal cell carcinoma, and inhibits cell growth, migration and invasion by targeting PTEN," (in eng), *Molecular medicine reports,* vol. 13, no. 6, pp. 4800-6, Jun 2016, doi: 10.3892/mmr.2016.5101.
[66] M. Friedrich *et al.*, "CREB1 is affected by the microRNAs miR-22-3p, miR-26a-5p, miR-27a-3p, and miR-221-3p and correlates with adverse clinicopathological features in renal cell carcinoma," (in eng), *Sci Rep,* vol. 10, no. 1, p. 6499, Apr 16 2020, doi: 10.1038/s41598-020-63403-y.
[67] V. Petrozza *et al.*, "Emerging role of secreted miR-210-3p as potential biomarker for clear cell Renal Cell Carcinoma metastasis," (in eng), *Cancer biomarkers : section A of Disease markers,* vol. 27, no. 2, pp. 181-188, 2020, doi: 10.3233/cbm-190242.
[68] H. Xiao and J. Shi, "Exosomal circular RNA_400068 promotes the development of renal cell carcinoma via the miR-210-5p/SOCS1 axis," (in eng), *Molecular medicine reports,* vol. 22, no. 6, pp. 4810-4820, Dec 2020, doi: 10.3892/mmr.2020.11541.

[69] C. Kalogirou *et al.*, "Identification of miR-21-5p and miR-210-3p serum levels as biomarkers for patients with papillary renal cell carcinoma: a multicenter analysis," (in eng), *Translational andrology and urology,* vol. 9, no. 3, pp. 1314-1322, Jun 2020, doi: 10.21037/tau.2020.03.18.

[70] Y. Chen, X. Wang, X. Zhu, and S. Shao, "Detection Performance of Circulating MicroRNA-210 for Renal Cell Carcinoma: a Meta-Analysis," (in eng), *Clinical laboratory,* vol. 64, no. 4, pp. 569-576, Apr 1 2018, doi: 10.7754/Clin.Lab.2017.171103.

[71] T. Lin, Y. Yang, X. Ye, J. Yao, and H. Zhou, "Low expression of miR-99b promotes progression of clear cell renal cell carcinoma by up-regulating IGF1R/Akt/mTOR signaling," (in eng), *International journal of clinical and experimental pathology,* vol. 13, no. 12, pp. 3083-3091, 2020.

[72] M. Lukamowicz-Rajska *et al.*, "MiR-99b-5p expression and response to tyrosine kinase inhibitor treatment in clear cell renal cell carcinoma patients," (in eng), *Oncotarget,* vol. 7, no. 48, pp. 78433-78447, Nov 29 2016, doi: 10.18632/oncotarget.12618.

[73] X. Xu, C. Liu, and J. Bao, "Hypoxia-induced hsa-miR-101 promotes glycolysis by targeting TIGAR mRNA in clear cell renal cell carcinoma," (in eng), *Molecular medicine reports,* vol. 15, no. 3, pp. 1373-1378, Mar 2017, doi: 10.3892/mmr.2017.6139.

[74] Y. Yamada *et al.*, "Replisome genes regulation by antitumor miR-101-5p in clear cell renal cell carcinoma," (in eng), *Cancer science,* vol. 111, no. 4, pp. 1392-1406, Apr 2020, doi: 10.1111/cas.14327.

[75] Y. Dong, Y. Gao, T. Xie, H. Liu, X. Zhan, and Y. Xu, "miR-101-3p Serves as a Tumor Suppressor for Renal Cell Carcinoma and Inhibits Its Invasion and Metastasis by Targeting EZH2," (in eng), *BioMed research international,* vol. 2021, p. 9950749, 2021, doi: 10.1155/2021/9950749.

[76] T. Sakurai *et al.*, "The enhancer of zeste homolog 2 (EZH2), a potential therapeutic target, is regulated by miR-101 in renal cancer cells," (in eng), *Biochemical and biophysical research communications,* vol. 422, no. 4, pp. 607-14, Jun 15 2012, doi: 10.1016/j.bbrc.2012.05.035.

[77] Y. Goto *et al.*, "The microRNA signature of patients with sunitinib failure: regulation of UHRF1 pathways by microRNA-101 in renal cell carcinoma," (in eng), *Oncotarget,* vol. 7, no. 37, pp. 59070-59086, Sep 13 2016, doi: 10.18632/oncotarget.10887.
